# Interferon regulatory factor 9 is critical for neointima formation following vascular injury

**DOI:** 10.1038/ncomms6160

**Published:** 2014-10-16

**Authors:** Shu-Min Zhang, Li-Hua Zhu, Hou-Zao Chen, Ran Zhang, Peng Zhang, Ding-Sheng Jiang, Lu Gao, Song Tian, Lang Wang, Yan Zhang, Pi-Xiao Wang, Xiao-Fei Zhang, Xiao-Dong Zhang, De-Pei Liu, Hongliang Li

**Affiliations:** 1 Department of Cardiology, Renmin Hospital of Wuhan University, Wuhan 430060, China; 2 Cardiovascular Research Institute, Wuhan University, Wuhan 430060, China; 3 State Key Laboratory of Medical Molecular Biology, Department of Biochemistry and Molecular Biology, Institute of Basic Medical Sciences, Chinese Academy of Medical Sciences, Peking Union Medical College, Beijing 100005, China; 4 Department of Cardiology, Institute of Cardiovascular Disease, Union Hospital, Tongji Medical College, Huazhong University of Science and Technology, Wuhan 430022, China; 5 College of Life Sciences, Wuhan University, Wuhan 430072, China

## Abstract

Interferon regulatory factor 9 (IRF9) has various biological functions and regulates cell survival; however, its role in vascular biology has not been explored. Here we demonstrate a critical role for IRF9 in mediating neointima formation following vascular injury. Notably, in mice, IRF9 ablation inhibits the proliferation and migration of vascular smooth muscle cells (VSMCs) and attenuates intimal thickening in response to injury, whereas IRF9 gain-of-function promotes VSMC proliferation and migration, which aggravates arterial narrowing. Mechanistically, we show that the transcription of the neointima formation modulator SIRT1 is directly inhibited by IRF9. Importantly, genetic manipulation of SIRT1 in smooth muscle cells or pharmacological modulation of SIRT1 activity largely reverses the neointima-forming effect of IRF9. Together, our findings suggest that IRF9 is a vascular injury-response molecule that promotes VSMC proliferation and implicate a hitherto unrecognized ‘IRF9–SIRT1 axis’ in vasculoproliferative pathology modulation.

## Abstract (web summary)

Blood vessels respond to injury by thickening the supportive smooth muscle layer in a process known as neointima formation. Here the authors describe a novel regulatory pathway of neointima formation that involves a transcription factor, Interferon Regulating Factor 9, and its downstream target, the deacetylase SIRT1.

## Graphical abstract

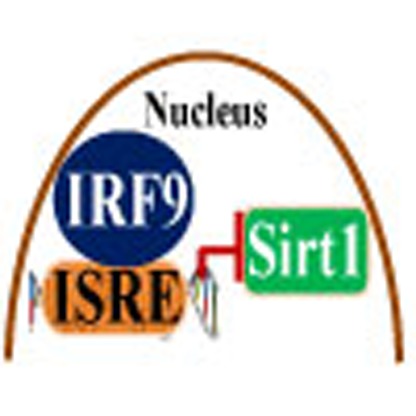

Arterial intimal hyperplasia is a prevalent severe pathophysiological process that contributes to the progression of atherosclerosis, in-stent restenosis and vein bypass graft failure^1^. In response to injury and other stimuli, vascular smooth muscle cells (VSMCs) proliferate, migrate and secrete extracellular matrix, thus forming a neointima^2^. During neointima formation, growth factors, such as transforming growth factor-β and platelet-derived growth factor (PDGF), are produced locally^3^, and vasculostabilizing factors, such as SIRT1, are attenuated^4^. Consequently, cell cycle genes, including proliferating cell nuclear antigen (PCNA) and Cyclin D1, are upregulated in VSMCs to facilitate cell growth^5^, and matrix metalloproteinases (MMPs) are highly expressed to facilitate VSMC migration from the media to the intima^6^. As a result, the blood vessels narrow or become occluded. The treatments aimed at suppressing neointima formation are extremely limited, however. Thus, further investigation into the pathophysiology and molecular mechanisms underlying neointima formation is urgently required.

Interferon (IFN) regulatory factors (IRFs) constitute a family of transcription factors with nine members (IRF1–IRF9) in mammals^7^. IRFs are well characterized as regulators of immunity and cell survival^8^,^9^,^10^. Accordingly, the ubiquitously expressed IRF9 mediates the effects of IFNs^11^. In response to IFN-α/β, IRF9 induces p53-dependent apoptosis, which indicates an antiproliferative role for IRF9 (ref. 12). Moreover, IRF9 was also implicated in antitumour drug resistance and the promotion of cell growth^13^. However, the role of IRF9 in VSMC-responsive proliferation remains enigmatic. In addition to functions in immunity and cell fate decision, our group recently revealed crucial roles for IRFs in the development of metabolic and heart diseases^14^,^15^,^16^,^17^,^18^,^19^. In particular, IRF9 was implicated in the regulation of hepatic steatosis, insulin resistance, cardiac hypertrophy and heart failure^16^,^19^. Hence, an investigation of the role of IRF9 in vascular biology and remodelling would improve our understanding of the intricate mechanisms of IRF9 action and hopefully inspire new strategies for treating many vascular diseases.

In this study, we determine a role for IRF9 in neointima formation. More importantly, we uncover a role for IRF9 as a negative transcriptional regulator of the vascular protective factor SIRT1 in response to injury. Correspondingly, modulating SIRT1 expression or its deacetylase activity completely reverses the neointima-forming effect of IRF9. Thus, our findings strongly suggest the existence of a previously unrecognized ‘IRF9–SIRT1 axis’ during vasculoproliferative pathology modulation.

## Results

### IRF9 increases in VSMCs during neointima formation

**Figure 1:**
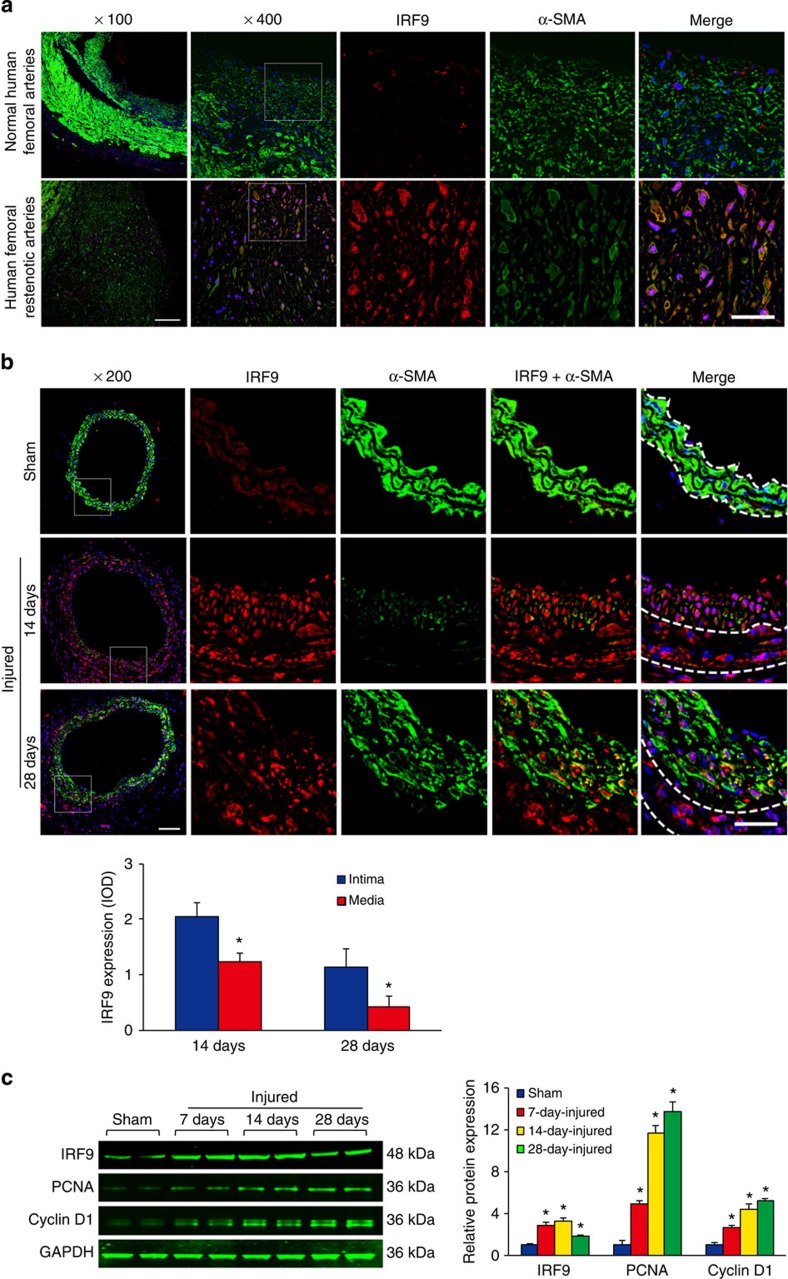
IRF9 expression is upregulated during neointima formation. (**a**) Immunofluorescence staining for IRF9 in a normal femoral artery (upper panels) and the neointima in an in-stent restenotic human femoral artery (lower panels). IRF9 is depicted in red, α-SMA is shown in green and 4',6-diamidino-2-phenylindole (DAPI) is presented in blue (*n*=3 per group). Scale bar, 50 μm. (**b**) Immunofluorescence staining shows IRF9 (red) expression and localization in the arteries at various time points post injury. The arterial smooth muscle cells are indicated by α-SMA (green). DAPI (blue) indicates nuclei. Scale bar, 50 μm. The IRF9 fluorescence relative OD values are also provided. (*n*=3 for sham group, *n*=4 for 14 and 28 days injured group, *P<0.05 versus intima). (**c**) The IRF9, PCNA and Cyclin D1 protein levels in the left carotid arteries were determined using western blot analysis (left). The arteries were harvested from uninjured (sham operation) and injured mice at 7, 14 or 28 days after wire injury. The protein levels were normalized to GAPDH. Right panel: the protein levels were quantified (*n*=3 samples at each time point, three to five carotid arteries were collected as a sample. *P<0.05 versus sham group). The blots represent three independent experiments. (**b**,**c**) The data represent the mean±s.d. Statistical analysis was carried out using one-way analysis of variance (ANOVA) test.

To investigate the involvement of IRF9 in neointima formation, we first examined its expression in human femoral artery specimens with or without in-stent restenosis. Using immunofluorescence staining, we observed that IRF9 expression was markedly increased in stenotic artery neointima compared with normal arteries (Fig. 1a). The increased IRF9 was primarily localized in the nuclei of neointima VSMCs, which were identified by α-smooth muscle actin (α-SMA) staining (Fig. 1a). To examine IRF9 expression in endothelial cells, we co-stained human specimens for CD31, an endothelial marker, and IRF9. We observed that some endothelial cells were destroyed after injury. In the remaining endothelial cells, IRF9 expression was minimal, and the expression levels were not significantly different between normal and restenotic arteries (Supplementary Fig. 1a). Subsequently, we treated rat aortic VSMCs (RASMCs) with platelet-derived growth factor-BB (PDGF-BB) and examined IRF9 expression. Before PDGF-BB treatment, IRF9 was primarily localized in the cytoplasm, whereas after PDGF-BB treatment IRF9 was primarily localized in the nucleus (Supplementary Fig. 1b). Western blot analysis revealed that IRF9 expression also increased after PDGF-BB stimulation (Supplementary Fig. 1c). These results indicate that IRF9 is a stress-responsive factor that is elevated and activated by PDGF-BB and translocates into the nucleus in VSMCs. To investigate whether IRF9 expression can be induced by vascular injury *in vivo*, we performed carotid artery wire injury surgery in mice (Supplementary Fig. 1d). Using immunofluorescence staining, we observed that IRF9 was primarily located in VSMCs, which were identified by α-SMA expression (Fig. 1b). The optical density (OD) of IRF9 in VSMCs increased at 14 days after injury and mildly decreased at 28 days after injury (Fig. 1b). The OD of IRF9 in neointima VSMCs was significantly higher compared with the media VSMCs at 14 days and 28 days after injury (Fig. 1b). Next, we co-stained IRF9 with CD31 to examine IRF9 expression in endothelial cells. Similar to the results in human specimens, we observed that minimal IRF9 was expressed in CD31^+^ cells in mouse arteries and that IRF9 expression in endothelial cells did not significantly differ between the sham-operated and wire injury-operated groups (Supplementary Fig. 1e). Immunofluorescence staining results revealed that the expressions of proliferation markers, namely PCNA and Cyclin D1, were upregulated, whereas expression of the differentiation marker α-SMA was reduced after wire injury (Supplementary Fig. 1f). Western blot results confirmed that the injury induced IRF9 expression (Fig. 1c). At 7 and 14 days after the injury, IRF9 expression was markedly increased compared with its level before injury (Fig. 1c). Twenty-eight days after the injury, IRF9 expression mildly declined as the acute phase elapsed (Fig. 1c). The above results indicate that IRF9 is upregulated and activated in VSMCs during neointima formation.

### IRF9 deficiency suppresses neointima formation

**Figure 2:**
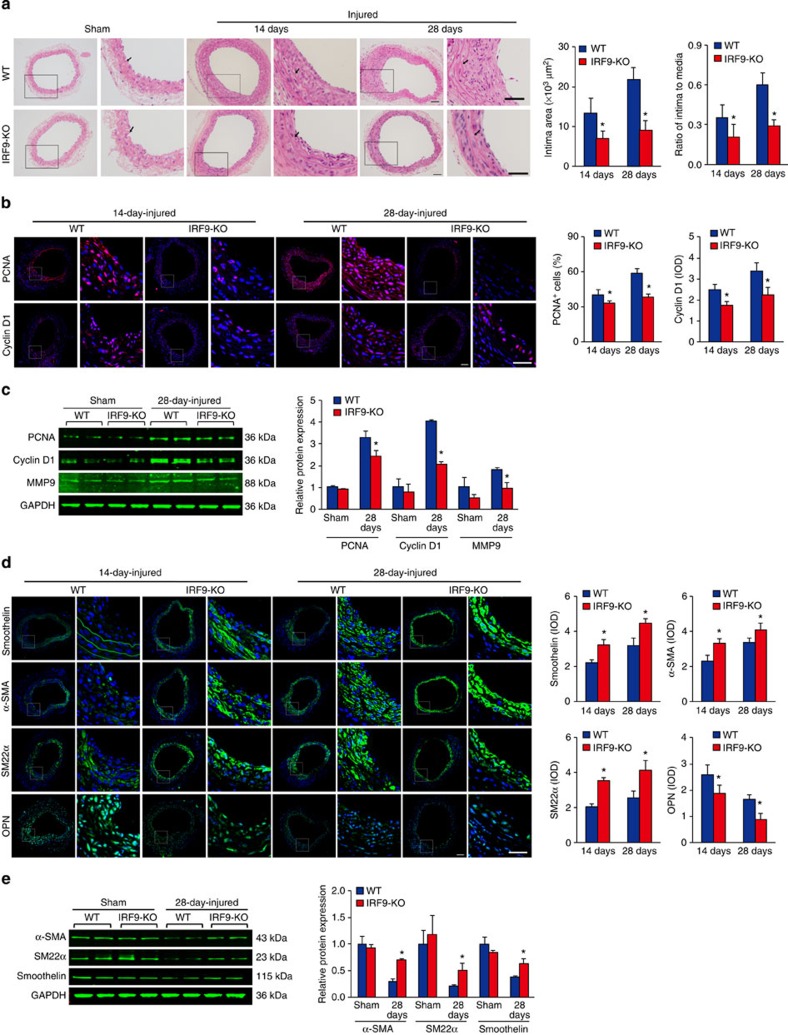
IRF9 deficiency suppresses neointima formation. (**a**) haematoxylin and eosin (HE)-stained sections show the carotid artery structures from WT and *IRF9*-KO mice that underwent a sham operation or14- and 28-day post wire injury. The insets in the left panels were magnified and are presented in the right panels. The black arrows indicate inner elastic discs. Scale bar, 50 μm. The intimal areas and intima/media ratios were quantified (*n*=7–12 at 14 days, *n*=8–12 at 28 days). (**b**) Immunofluorescence staining shows PCNA and Cyclin D1 (red) expression and localization in WT and *IRF9*-KO carotid arteries at 14 and 28 days post injury. DAPI (blue) indicates the nuclei. Scale bar, 50 μm. The proportion of PCNA-positive cells and Cyclin D1 OD levels were calculated (*n*=3–5 at 14 days, *n*=4–5 at 28 days). (**c**) Western blot analysis (left) and quantification (right) of the PCNA, Cyclin D1 and MMP9 protein levels in arteries from WT and *IRF9*-KO mice with sham operation or 28-day-post injury (*n*=3 samples per group at each time point, three to four carotid arteries were collected as a sample). The blots represent three independent experiments. (**d**) Immunofluorescence staining for Smoothelin, α-SMA, SM22α and OPN (green in each row) in WT and *IRF9*-KO arteries at 14 and 28 days after injury. DAPI (blue) staining indicates the nuclei. Scale bar, 50 μm. The OD values for smoothelin, α-SMA, SM22α and OPN fluorescence are also provided (*n*=4–6 per group at 14 days, *n*=3–6 per group at 28 days). (**e**) α-SMA, SM22α and Smoothelin protein levels in arteries from WT and *IRF9*-KO mice with sham operation or 28 days post injury were determined using western blot analysis (left). The expression levels were normalized to GAPDH and quantified (right, *n*=3 samples per group at each time point, three to four carotid arteries were collected as a sample). The blots represent three independent experiments. (**a**–**e**) The values are presented as the mean±s.d., *P<0.05 versus the WT group. Statistical analysis was carried out using independent sample *t*-test.

IRF9 induction in VSMCs upon vascular injury suggests a possible regulatory role for IRF9 in neointima formation. Hence, we performed carotid artery wire injury surgery on IRF9 global knockout (*IRF9*-KO) mice and wild-type (WT) controls to investigate this hypothesis. The absence of IRF9 in the arteries of *IRF9*-KO mice was verified with immunofluorescence staining (Supplementary Fig. 2). The neointima in the *IRF9*-KO mice was significantly thinner compared with the WT mice (Fig. 2a). The intima-to-media (I/M) ratio in the *IRF9*-KO arteries was only approximately half the ratio observed in the WT controls at 14 and 28 days post injury (Fig. 2a). Immunofluorescence staining and western blot analyses revealed that the expression levels of the proliferative genes Cyclin D1 and PCNA were downregulated in *IRF9*-KO mice (Fig. 2b,c). The migration of media-resident VSMCs to the intima contributes to neointima formation^6^. The western blot results revealed that MMP9 expression was inhibited in *IRF9*-KO mice, which indicates constrained cell migration (Fig. 2c). To facilitate proliferation and migration in response to injury and other stimuli, contractile smooth muscle cells (SMCs) are transformed into synthetic SMCs in a process that is referred to as phenotypic switching^3^. Immunofluorescence staining and western blot analyses revealed that the expression of SMC-specific genes (*α-SMA*, *SM22α* and *Smoothelin*) increased and that osteopontin (OPN) expression decreased in the arteries of *IRF9*-KO mice, indicating that IRF9 deficiency attenuated VSMC phenotypic switching (Fig. 2d,e). Collectively, these data indicate that IRF9 deficiency suppresses neointima formation.

### IRF9 overexpression in SMC promotes neointima formation

**Figure 3:**
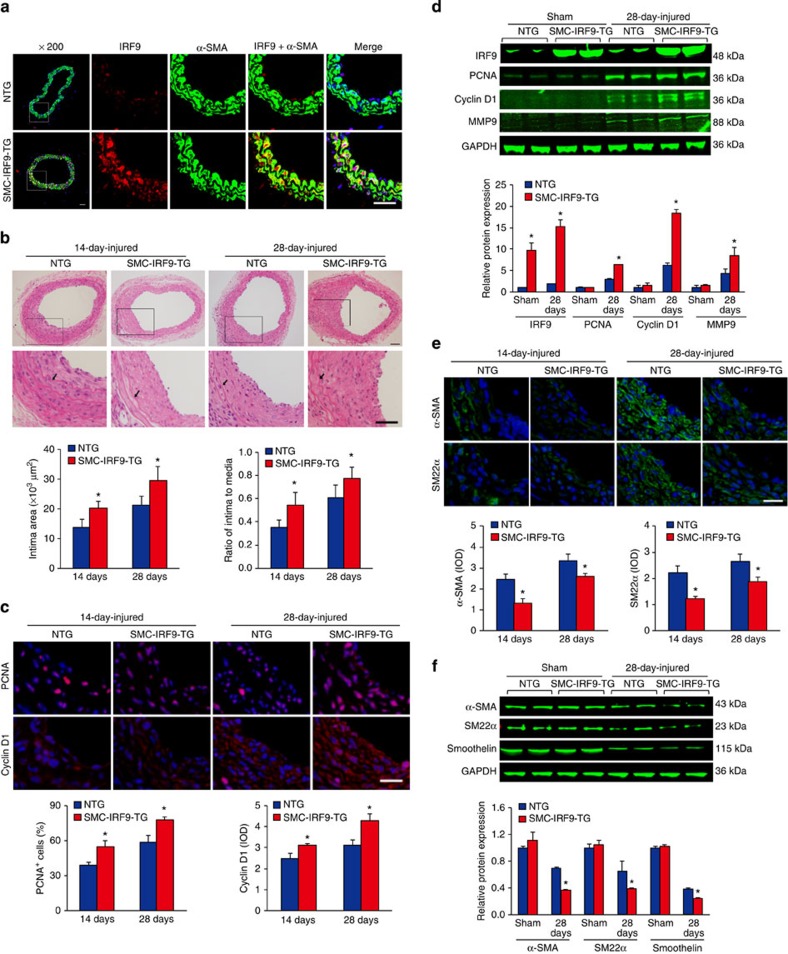
SMC-specific IRF9 overexpression promotes neointima formation. (**a**) Immunofluorescence staining of IRF9 (red), α-SMA (green) and DAPI (blue) shows the effectiveness and specificity of IRF9 overexpression. Scale bar, 50 μm. (*n*=3 per group). (**b**) The HE-stained sections show the NTG and SMC-*IRF9*-TG carotid arterial structures at 14 or 28 days post injury. Insets in the upper panels were magnified and are presented in the lower panels. The black arrows indicate inner elastic discs. Scale bar, 50 μm. The intimal areas and intima/media ratios were quantified (*n*=6–14 at 14 days, *n*=6–14 at 28 days). (**c**) Immunofluorescence staining shows PCNA and Cyclin D1 (red) expression and localization in the carotid arteries of NTG and SMC-*IRF9*-TG mice at 14 and 28 days post injury. DAPI (blue) indicates the nuclei. Scale bar, 50 μm. The proportion of PCNA-positive cells and the OD values of Cyclin D1 were calculated (*n*=3 at 14 days, *n*=3–6 at 28 days). (**d**) The IRF9, PCNA, Cyclin D1 and MMP9 protein levels in the NTG and SMC-*IRF9*-TG arteries with sham operation or 28 days post injury were measured using western blot analysis. (*n*=3 samples per group at each time point, three to four carotid arteries were collected as a sample). (**e**) Immunofluorescence staining for α-SMA and SM22α (green in different rows) in NTG and SMC-*IRF9*-TG arteries at 14 and 28 days after injury. DAPI (blue) staining indicates the nuclei. Scale bar, 20 μm. The OD values for α-SMA and SM22α fluorescence are also provided (*n*=3–6 at 14 days, *n*=3–4 at 28 days). (**f**) The α-SMA, SM22α and Smoothelin protein levels in the NTG and SMC-*IRF9*-TG arteries with sham operation or 28-day-post injury were measured using western blot analysis (*n*=3 samples per group at each time point, three to four carotid arteries were collected as a sample). (**d**,**f**) The expression levels were normalized to GAPDH and quantified. The blots represent three independent experiments. (**b**–**f**) The values are presented as the mean±s.d, *P<0.05 versus the NTG group. Statistical analysis was carried out using independent sample *t*-test.

Given that IRF9 is induced in VSMCs upon vascular injury and that intima growth is inhibited in *IRF9*-KO mice, we next questioned whether IRF9 overexpression would facilitate neointima formation. To eliminate the possible influence of cell types other than VSMCs, we generated a strain of SMC-specific IRF9 transgenic (SMC-*IRF9*-TG) mice in the C57BL/6J background. The transgenic construct included mouse IRF9 cDNA controlled by an SMC-specific mouse minimal *SM22α* promoter (Supplementary Fig. 3a). Four SMC-*IRF9*-TG mouse lines were successfully generated; line 4 exhibited the highest (4.1-fold) increase in IRF9 expression (Supplementary Fig. 3b), which was comparable to the level in WT arteries at 14 days post injury. Therefore, this line was used in the subsequent phenotypic evaluations. To validate that the SMC-*IRF9*-TG line is functional and specific *in vivo*, we co-stained IRF9 with α-SMA and CD31, respectively, in the arteries. We observed that almost all of the IRF9 localized in α-SMA^+^ cells. Furthermore, IRF9 expression in α-SMA^+^ cells in the TG group were significantly increased compared with the non-transgenic (NTG) group (Fig. 3a). By contrast, only minimal IRF9 expression was observed in CD31^+^ cells, and these levels were not significantly different between the NTG and TG groups at baseline (Supplementary Fig. 3c). Thus, IRF9 overexpression in SMC-*IRF9*-TG mice is effective and SMC-specific. At 14 and 28 days after arterial injury, the area of the newly formed intima and the I/M ratio were increased in the SMC-*IRF9*-TG mice compared with the NTG mice (Fig. 3b). Similar to the results in line 4 TG mice, the exacerbated neointima areas were also observed in the injured arteries of lines 1 and 3 SMC-*IRF9*-TG mice compared with NTG controls (Supplementary Fig. 3d). Cyclin D1, PCNA and MMP9 expression levels were increased and SMC-specific genes (*α-SMA*, *SM22α* and *Smoothelin*) expression levels were decreased in the IRF9-overexpressing arteries, as determined by immunofluorescence staining and western blotting (Fig. 3c–f). Considering that IRF9 is induced in VSMCs upon vascular injury, IRF9 appears to be a genuine mediator of neointima formation.

### IRF9 promotes VSMC proliferation and migration

**Figure 4:**
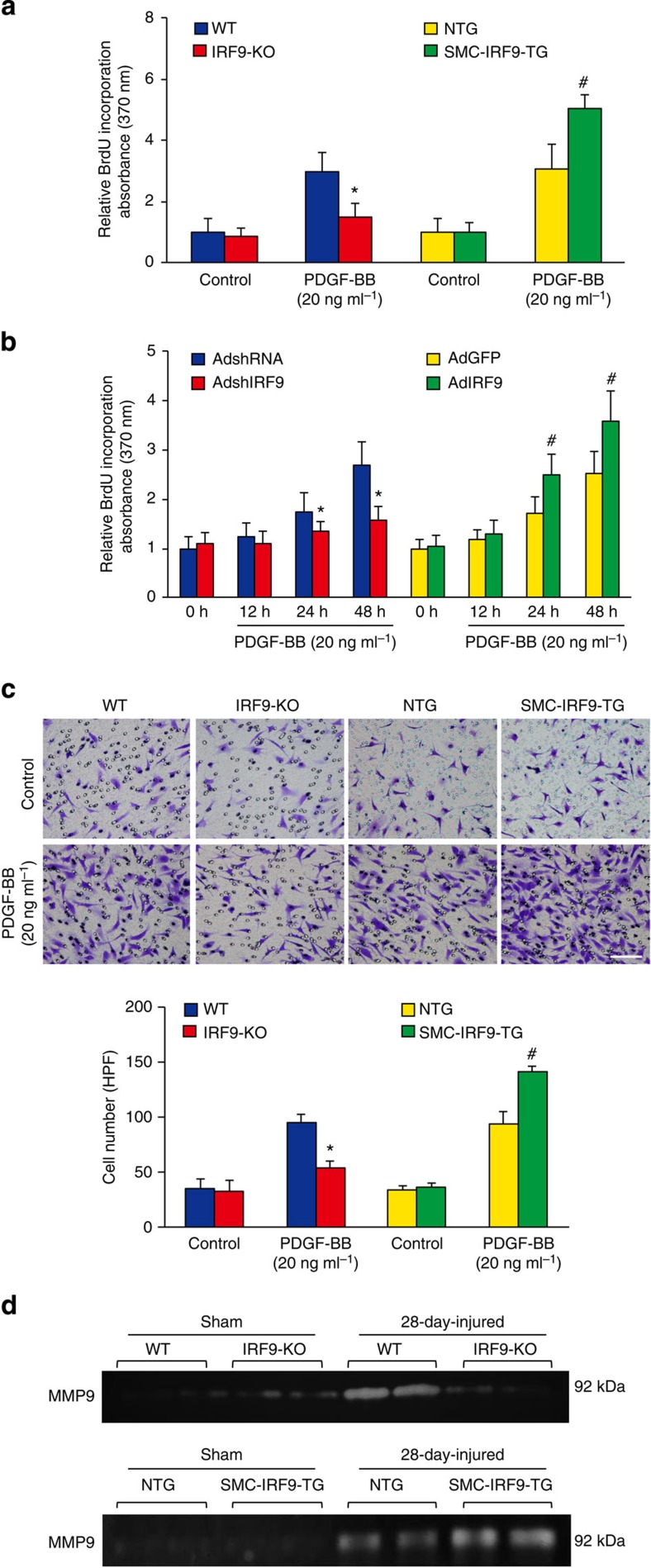
IRF9 promotes VSMC proliferation, migration and phenotypic switching. (**a**,**b**) BrdU incorporation was determined by measuring the absorption at 370 nm to assess SMC proliferation upon PDGF-BB stimulation. (**a**) *IRF9*-KO and WT primary mouse VSMCs and SMC-*IRF9*-TG and NTG primary mouse VSMCs were harvested before or 24 h after PDGF-BB administration (*n*=8, *P<0.05 versus the WT group; ^#^*P*<0.05 versus the NTG group). (**b**) The RASMCs were infected with AdshRNA and AdshIRF9 adenoviruses as well as AdGFP and AdIRF9 adenoviruses 24 h before PDGF-BB administration. The cells were harvested before or at 12, 24 or 48 h after PDGF-BB administration (*n*=5, *P<0.05 versus the AdshRNA-infected group; ^#^*P*<0.05 versus the AdGFP-infected group). (**c**) Primary mouse VSMCs were seeded on the transwell dishes to perform migration assays. PDGF-BB was used as a chemoattractant. Cells migrating through the membrane were fixed after 6-h PDGF-BB or PBS treatment. Scale bar, 100 μm. The cell number per high-power field was counted (*n*=7, *P<0.05 versus the WT group; ^#^*P*<0.05 versus the NTG group). (**d**) Fresh carotid arteries from sham-operated and 28-day-injured mice were collected to perform gelatin zymography to examine MMP9 activity (*n*=3 samples per group at each time point, three to four carotid arteries were collected as a sample). (**a**–**d**) The values are presented as the mean±s.d. The results represent three independent experiments. Statistical analysis was carried out using independent sample *t*-test.

We observed that IRF9 mediated neointima formation induced by vascular injury. Thus, to confirm these *in vivo* results, we isolated and cultured primary VSMCs from *IRF9*-KO and SMC-*IRF9*-TG mice and subsequently mimicked the injury response *in vitro* by treating these cells with PDGF-BB. We evaluated SMC proliferation by measuring 5′-bromo-2′-deoxyuridine (BrdU) incorporation. After PDGF-BB stimulation, *IRF9*-KO VSMCs incorporated less BrdU, whereas SMC-*IRF9*-TG cells incorporated more BrdU than their corresponding WT control cells (Fig. 4a), which indicates that IRF9 facilitates a proliferative response upon PDGF-BB stimulation. We then modulated IRF9 levels by knocking down or overexpressing IRF9 in primary RASMCs and subsequently examining BrdU incorporation. We observed that the amount of BrdU incorporation was positively related to IRF9 expression in VSMCs (Fig. 4b). Finally, we modulated IRF9 levels in human aortic SMCs (HASMCs) and examined BrdU incorporation. Consistent with the results in mouse primary SMCs and in RASMCs, IRF9 facilitated a proliferative response upon PDGF-BB stimulation in HASMCs (Supplementary Fig. 4a–e).

Next, to evaluate SMC migration, we used a modified Boyden chamber migration assay. The VSMC migration assay revealed that after PDGF-BB (20 ng ml^−1^) stimulation, fewer *IRF9*-KO VSMCs migrated through the membrane than WT VSMCs; however, IRF9 overexpression significantly increased PDGF-BB-induced migration (Fig. 4c). Furthermore, a gelatin zymography assay indicated that MMP9 activity in the *IRF9*-KO group was significantly reduced compared with the WT group at 28 days post injury, whereas MMP9 activity in the SMC-*IRF9*-TG group was notably increased compared with the NTG group, indicating that IRF9 facilitates SMC migration (Fig. 4d). Consistently, western blot analyses revealed that VSMC proliferation, migration and phenotypic switching upon PDGF-BB stimulation were suppressed through *IRF9*-KO, whereas IRF9 overexpression enhanced this response (Supplementary Fig. 5a,b). Using both *in vivo* and *in vitro* experiments, we determined that IRF9 promotes VSMC proliferation, migration and subsequently neointima formation in a cell-autonomous manner.

### IRF9 suppresses SIRT1 transcription

**Figure 5:**
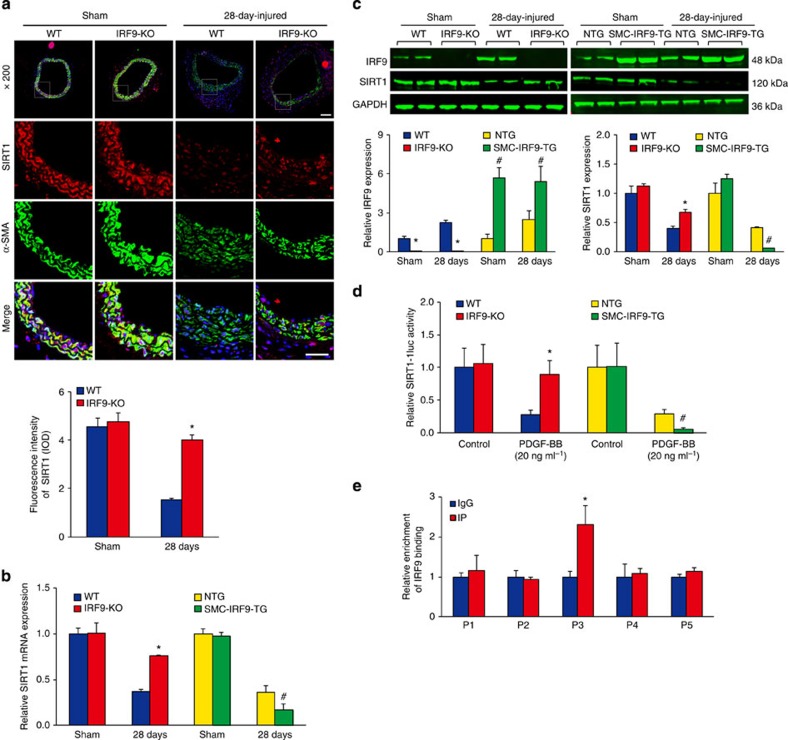
IRF9 suppresses SIRT1 transcription. (**a**–**c**) Mice underwent a sham or wire injury operation, and 28 days later, their carotid arteries were collected to examine SIRT1 expression. (**a**) Immunofluorescence staining for SIRT1 (red) and α-SMA (green) was performed to determine SIRT1 localization. The corresponding OD values are also presented (*n*=3 for sham group, *n*=3–4 for 28-day-injured group, *P<0.05 versus the WT group). (**b**) Using real-time PCR, we measured SIRT1 mRNA levels in the WT and *IRF9*-KO and in the NTG and SMC-*IRF9*-TG samples (*n*=4 per group at each time point). (**c**) The IRF9 and SIRT1 protein levels were determined using western blot analysis. The expression levels were normalized to GAPDH and quantified (*n*=5 per group at each time point). The blots shown are representative of three independent experiments. (**d**) Adenovirus constructs with SIRT1-luciferase were used to infect WT, *IRF9*-KO, SMC-*IRF9*-TG and NTG primary mouse VSMCs. The cells were treated with PDGF-BB (20 ng ml^−1^) for 24 h or were untreated before the luciferase activity was measured (*n*=9 per group). The result represents three independent experiments. (**b**–**d**) *P<0.05 versus the WT group; ^#^*P*<0.05 versus the NTG group. (**e**) ChIP experiments were performed to measure the relative enrichment of IRF9 binding at the five putative ISREs (*n*=3 per group). *P<0.05 versus IgG group. The result represents three independent experiments. (**a**–**e**) The values are presented as the mean±s.d. Statistical analysis was carried out using independent sample *t*-test.

The evident neointima-forming effect of IRF9 prompted us to investigate its underlying mechanism. A genome-wide microarray screen and ingenuity pathway analysis revealed that the mRNA levels of proliferation- and migration-related genes, such as *MMP9*, *Cyclin D1*, *c-Fos* and *c-Jun*, were significantly increased in the carotid arteries of SMC-*IRF9*-TG mice compared with the NTG controls at 14 days post injury (Supplementary Table 1). Our previous study determined that SIRT1 inhibits AP-1-dependent Cyclin D1 and MMP9 transcriptions, thereby suppressing VSMC proliferation and migration and preventing neointima formation^4^. Interestingly, SIRT1 and SIRT1-dependent molecular targets were also markedly altered in the SMC-*IRF9*-TG mice compared with NTG mice in response to wire injury (Supplementary Table 1). More importantly, real-time PCR further confirmed these results, indicating that SIRT1 plays crucial roles in the neointima formation induced by IRF9 overexpression (Supplementary Fig. 6). In agreement with our previous report, SIRT1 expression was decreased at both the protein and mRNA levels in the VSMCs of WT mice following arterial injury^4^ (Fig. 5a–c). Notably, the reduction in SIRT1 expression in VSMCs after injury was markedly reversed in *IRF9*-KO mice, whereas the SIRT1 reduction was enhanced in SMC-*IRF9*-TG mice (Fig. 5a–c). These findings indicate that IRF9 may inhibit SIRT1 expression. In view of this possibility, we introduced different doses of the IRF9-Myc plasmid into the mouse VSMC line MOVAS and subsequently examined SIRT1 promoter activity using luciferase reporter assays. We observed that when the IRF9-Myc levels increased, SIRT1 promoter activity was suppressed to a greater extent (Supplementary Fig. 7a). We then treated *IRF9*-KO, SMC-*IRF9*-TG and WT primary mouse VSMCs with PDGF-BB and examined SIRT1 promoter activity. As expected, SIRT1 promoter activity decreased upon PDGF-BB stimulation in WT cells; this reduction in activity was blocked in *IRF9*-KO cells but was more prominent in SMC-*IRF9*-TG cells (Fig. 5d). These results indicate that SIRT1 expression is suppressed by IRF9.

Considering that IRF9 primarily acts as a transcription factor, we reasoned that IRF9 may modulate neointima formation through the direct suppression of SIRT1 transcription. To investigate this possibility, we performed chromatin immunoprecipitation (ChIP) of Myc–IRF9 in the mouse VSMC line MOVAS followed by quantitative PCR of the SIRT1 promoter (−2,500 to +200 bp around the transcription start site; Supplementary Fig. 7b). To increase the specificity of the ChIP analysis, sequences containing at least two IRF-stimulated response element (ISRE) repeats (5′-GAAA-3′) were considered as potential IRF9-binding sites. Using bioinformatics approaches, a series of putative ISRE-binding sites, designated P1–P5, were identified. We observed that IRF9 ChIPs were enriched for the P3 region but not any of the other regions, indicating that P3 contains the primary site for IRF9 binding (Fig. 5e). To further validate the importance of this binding site, the 5′-GAAA-3′ sequence in the IRF9-binding site P3 region was mutated to generate a mutant murine SIRT1 promoter (Mu-mSIRT1-luc). As expected, IRF9 retained the ability to increase the promoter activity of the WT (WT-mSIRT1-luc) but not mutant SIRT1 promoter, underscoring the importance and necessity of this binding site (Supplementary Fig. 7c).

To verify that SIRT1 is a genuine downstream target of IRF9, we modulated SIRT1 levels in different primary mouse VSMCs and examined cell proliferation. As mentioned earlier, BrdU incorporation induced by PDGF-BB stimulation was mitigated in *IRF9*-KO VSMCs, whereas its incorporation was enhanced in SMC-*IRF9*-TG VSMCs (Supplementary Fig. 7d). The induction of BrdU incorporation was maintained by shSIRT1 interference targeting SIRT1 in *IRF9*-KO VSMCs, whereas this induction was abrogated by SIRT1 overexpression in SMC-*IRF9*-TG VSMCs (Supplementary Fig. 7d). Taken together, these results indicate that IRF9 directly inhibits SIRT1 transcription to promote VSMC proliferation.

### The neointima-forming effect of IRF9 is mediated by SIRT1

**Figure 6:**
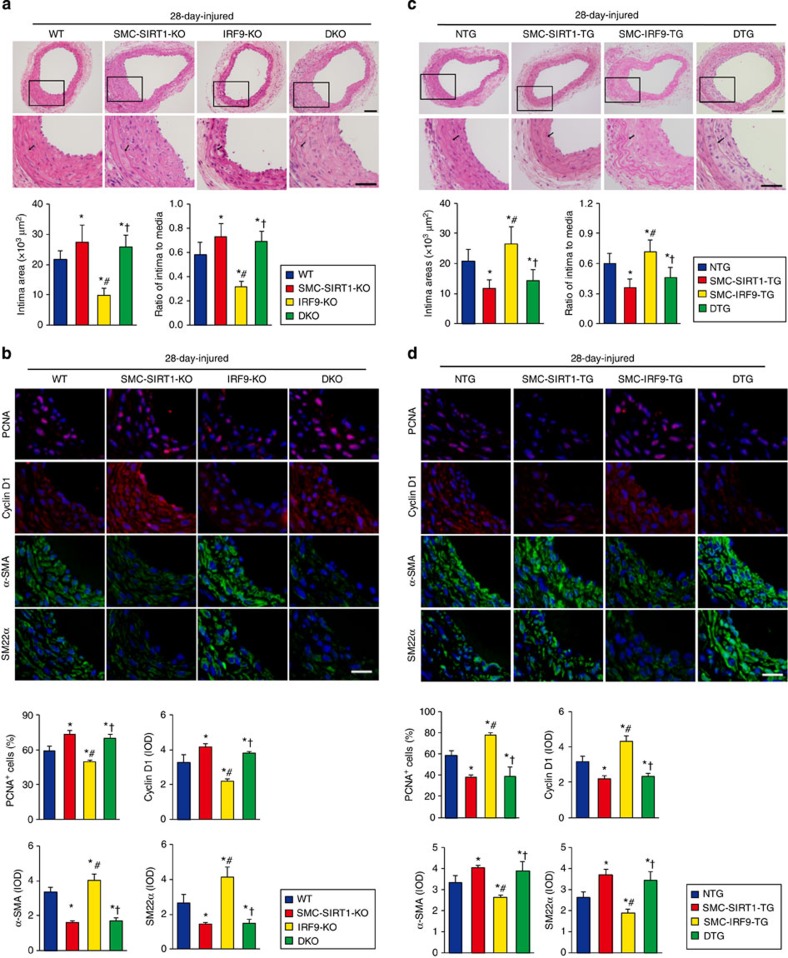
The effect of IRF9 on neointima formation is mediated by SIRT1. (**a**,**c**) HE-stained sections show the structures of carotid arteries from WT, SMC-*SIRT1*-KO, *IRF9*-KO and DKO mice (**a**) or NTG, SMC-*SIRT1*-TG, SMC-*IRF9*-TG and DTG mice (**c**) at 28 days post injury. The insets in the upper panels were magnified, and the magnified images are presented in the lower panels. The black arrows indicate inner elastic discs. Scale bar, 50 μm. The intimal areas and intima/media ratios were quantified (**a**, *n*=5–12 per group, **c**, *n*=6–10 per group). (**b**,**d**) Immunofluorescence staining shows PCNA, Cyclin D1 (red), α-SMA and SM22α (green) expression and localization in carotid arteries of WT, SMC-*SIRT1*-KO, *IRF9*-KO and DKO mice (**b**) or NTG, SMC-*SIRT1*-TG, SMC-*IRF9*-TG and DTG mice (**d**) at 28 days post injury. DAPI (blue) staining indicates the nuclei. Scale bar, 20 μm. The proportion of PCNA-positive cells and OD values for Cyclin D1, α-SMA and SM22α were calculated ((**b**) *n*=3–5 per group, (**d**) *n*=3–6 per group). (**a**–**d**) The values are presented as the mean±s.d., *P<0.05 versus the WT or NTG group, ^#^*P*<0.05 versus the SMC-*SIRT1*-KO group or the SMC-*SIRT1*-TG group, ^†^*P*<0.05 versus the *IRF9*-KO group or the SMC-*IRF9*-TG group. Statistical analysis was carried out using one-way ANOVA test.

Given that SIRT1 is a direct downstream target of IRF9, we next sought to examine whether SIRT1 mediates the effect of IRF9 on neointima formation *in vivo*. To accomplish this goal, we crossed *IRF9*-KO mice with SMC-specific *SIRT1*-KO (SMC-*SIRT1*-KO) mice (generated by crossing *Sirt1*^*flox/flox*^ mice with *SM22*-*Cre* mice) to generate SIRT1/IRF9 double knockout (DKO) mice (Supplementary Fig. 8a,b). We performed carotid artery wire injury surgery on the SMC-*SIRT1*-KO, *IRF9*-KO and DKO mice as well as the WT controls. Twenty-eight days after injury, the extent of neointima formation was examined. SMC-*SIRT1*-KO significantly aggravated neointima formation, even when IRF9 was also ablated (Fig. 6a). Consistent with the alterations in intima area and I/M ratio, SMC-*SIRT1*-KO abolished the reduction in PCNA and Cyclin D1 expression and the preservation of α-SMA and SM22α expression in the *IRF9*-KO vessels (Fig. 6b). Therefore, SIRT1 deficiency abolished the suppressive effect of IRF9 deficiency on neointima formation.

Given that SIRT1 ablation overcomes the vasculoprotective effect of IRF9 deficiency, we questioned whether a gain of SIRT1 expression could rescue the neointima-forming effect of IRF9 overexpression. We therefore simultaneously overexpressed SIRT1 and IRF9 in VSMCs by crossing SMC-specific *SIRT1*-TG (SMC-*SIRT1*-TG) and SMC-*IRF9*-TG mice (Supplementary Fig. 8c). We observed that the intima areas in *SIRT1/IRF9* double transgenic (DTG) mice were significantly smaller than those in the WT mice and comparable to those in the SMC-*SIRT1*-TG mice (Fig. 6c); thus, the neointima-promoting effect of IRF9 overexpression can be rescued by SIRT1 overexpression. Accordingly, the changes in PCNA, Cyclin D1, α-SMA and SM22α expression in the SMC-*IRF9*-TG arteries were also reversed by SIRT1 overexpression (Fig. 6d). On the basis of these findings, we conclude that SIRT1 reduction is required for IRF9-mediated neointima formation.

### The effects of IRF9 are dependent on SIRT1 activity

**Figure 7:**
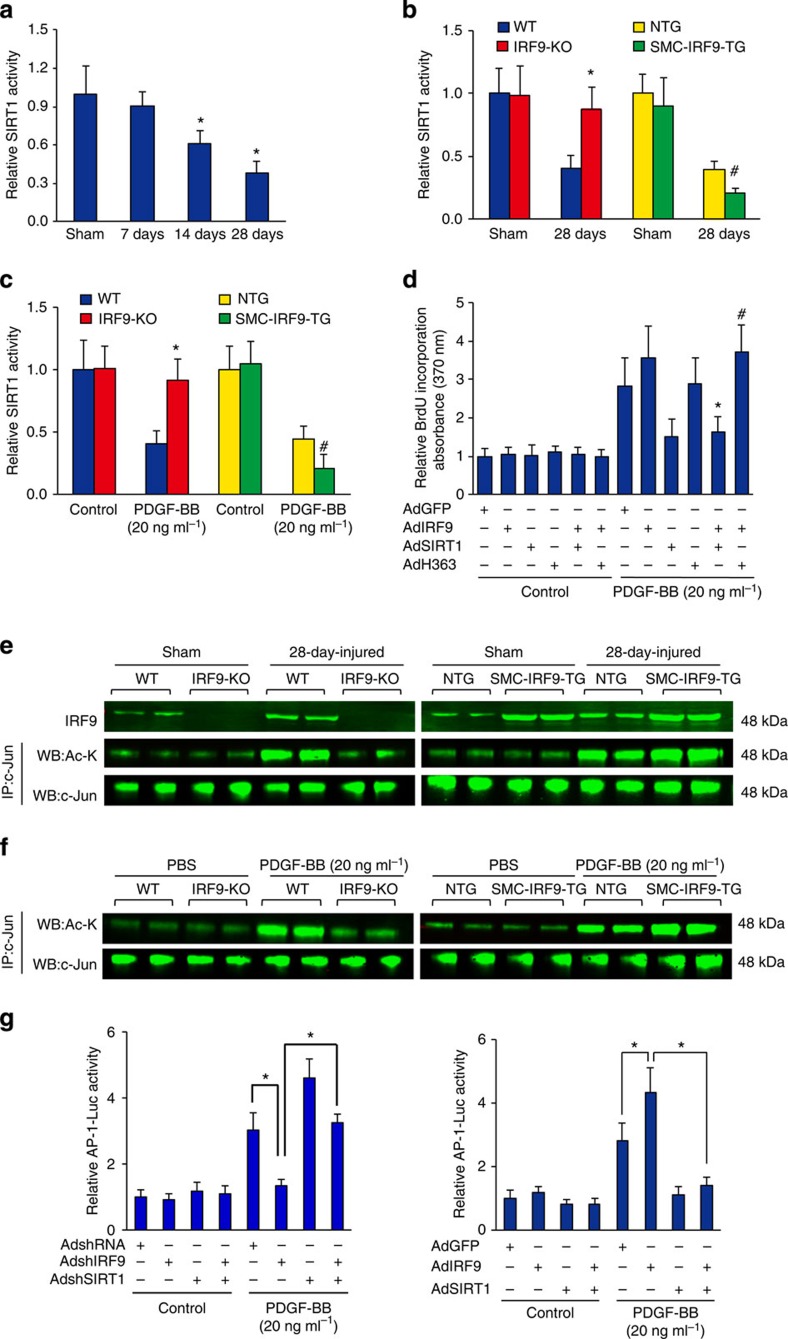
IRF9 inhibits AP-1 transactivation by suppressing SIRT1 transcription. (**a**) SIRT1 deacetylase activity in the carotid arteries of WT mice that underwent a sham operation or wire injury (7, 14 or 28 days after injury) was examined (*n*=4 per group at each time point, *P<0.05 versus sham group). (**b**) SIRT1 activity in the arteries of WT, *IRF9*-KO, SMC-*IRF9*-TG and NTG mice that underwent a sham or 28-day-injury operation (*n*=6–8 for sham group, *n*=6–8 for 28-day-injured group). (**c**) SIRT1 activity of WT and *IRF9*-KO primary mouse VSMCs and SMC-*IRF9*-TG and NTG primary mouse VSMCs that were treated with PDGF-BB (20 ng ml^−1^) for 24 h or left untreated (*n*=9–10 per group). (**b**,**c**) *P<0.05 versus the WT group; ^#^*P*<0.05 versus the NTG group. (**d**) BrdU labelling was used to evaluate cell proliferation before and after PDGF-BB stimulation. AdGFP, AdIRF9, AdSIRT1 and AdH363 (encoding the dominant-negative SIRT1 allele with His363 mutated to Tyr) were used to infect the RASMCs. *P<0.05 versus the AdIRF9-infected group, ^#^*P*<0.05 versus the AdIRF9/AdSIRT1-infected group. (*n*=9 per group). (**e**,**f**) The protein levels of IRF9, acetyl-c-Jun and total c-Jun were determined with western blot analysis to evaluate SIRT1 deacetylase activity. Sham-operated and injured arteries (**e**) and PBS or PDGF-BB-treated VSMCs (20 ng ml^−1^ for 6 h; **f**) were used. (*n*=3 samples per group at each time point, three to four carotid arteries were collected as a sample). (**g**) AP-1 (binding element)-luc was used to examine AP-1 transactivation. To manipulate IRF9 and SIRT1 levels, AdshIRF9, AdshSIRT1, AdIRF9 and AdSIRT1 were used to infect RASMCs. PDGF-BB was also used (*n*=12 per group). Statistical significance is indicated: *P<0.05; NS (not significant): *P*≥0.05. (**a**–**g**) The results represent three independent experiments. (**a**–**d**,**g**) the values are presented as the mean±s.d. Statistical analysis was carried out using independent sample *t*-test (**a**–**c**) or the one-way ANOVA test (**g**).

We found that genetic manipulation of SIRT1 effectively reverses neointima formation mediated by IRF9. SIRT1 is a class III deacetylase, and its effects are dependent on its enzymatic activity; therefore, we examined SIRT1 deacetylase activity in the arteries in our injury model. A decline in SIRT1 activity was observed after injury (Fig. 7a). Consistent with SIRT1 expression levels, SIRT1 deacetylase activity in mouse arteries and primary mouse VSMCs was also inversely related to IRF9 expression under the stimulation of wire injury or PDGF-BB (Fig. 7b,c). SIRT1 overexpression abolished the increase in BrdU incorporation triggered by IRF9 overexpression; however, when a dominant-negative SIRT1 (H363Y), which lacks deacetylase activity, was overexpressed, the increase in BrdU incorporation was unaltered (Fig. 7d). This finding indicates that the suppression of SIRT1 deacetylase activity is crucial for IRF9-mediated VSMC proliferation.

Our recent work has demonstrated that SIRT1 acts as a modulator of neointima formation in response to vascular injury through the regulation of AP-1-dependent targets^4^. Therefore, we then examined the status of AP-1 (c-Jun) deacetylation in different groups following vascular injury as well as in cultured vascular cells. At 28 days after injury in WT mice, c-Jun was acetylated. In contrast, *IRF9*-KO mice displayed a significant decrease in the expression of acetyl-c-Jun compared with WT mice subjected to wire injury (Fig. 7e), an effect that was reversed by the VSMC-specific overexpression of IRF9 *in vivo* (Fig. 7e). Our *in vitro* results also confirmed these findings (Fig. 7f).

To further confirm the effects of IRF9 on AP-1 activity, we examined whether IRF9 regulates the transcriptional activity of AP-1. We performed luciferase assays using an AP-1-luc reporter in primary cultured VSMCs. The cells were infected with AdIRF9 or AdshIRF9 and co-infected with or without AdSIRT1 or AdshSIRT1; the cells were then treated with PDGF-BB. PDGF-BB markedly stimulated AP-1 reporter activity, which was inhibited by infection with AdshIRF9 and further enhanced by infection with AdIRF9 (Fig. 7g). To assess whether IRF9-mediated AP-1 activity is SIRT1-dependent, we infected primary cultured vascular smooth cells with AdshIRF9, AdshSIRT1 or both AdshIRF9 and AdshSIRT1 (Fig. 7g). Co-infection with AdshSIRT1 largely abolished the protective effect of IRF9 inhibition (Fig. 7g). Conversely, the IRF9-mediated increase in AP-1 activity was reversed by AdSIRT1 (Fig. 7g). These data suggest that IRF9-mediated AP-1 activity is dependent on SIRT1 signalling.

As the transcription factor AP-1 is important in the regulation of Cyclin D1 and MMP9 transcription^4^, we performed a transient transfection analysis to determine whether IRF9 regulates Cyclin D1 and MMP9 transcription. Using two luciferase reporter vectors under the control of the Cyclin D1 (−903 to +202) and MMP9 (−711 to +19) promoters, we found that PDGF-BB significantly increased the activity of the Cyclin D1 and MMP9 promoters and that these increases were blocked by AdshIRF9 and promoted by AdIRF9 (Supplementary Fig. 9a,b). Importantly, co-infection with AdSIRT1 largely abolished the promoting effects of IRF9 on Cyclin D1 and MMP9 promoter activities (Supplementary Fig. 9b). Furthermore, point mutations or deletions of the AP-1 DNA-binding site in the Cyclin D1 and MMP9 promoters significantly impaired the ability of IRF9 to augment the PDGF-BB-induced activity of these promoters (Supplementary Fig. 9c,d).

**Figure 8:**
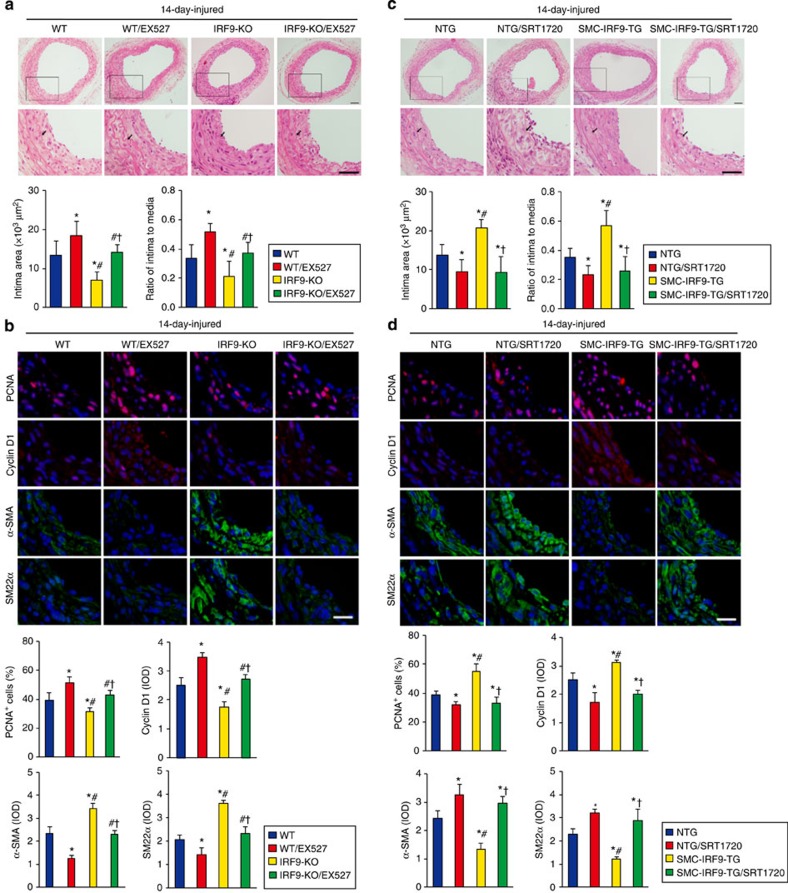
IRF9-mediated neointima formation is dependent on SIRT1 deacetylase activity. (**a**) HE-stained sections show the structure of carotid arteries of WT and *IRF9*-KO mice treated with EX527 or untreated at 14 days post injury. The insets in the upper panels were magnified, and the magnified images are presented in the lower panels. The black arrows indicate inner elastic discs. Scale bar, 50 μm. The intimal areas and intima/media ratios were quantified (*n*=5–12 per group). (**b**) Immunofluorescence staining shows PCNA and Cyclin D1 (red) as well as α-SMA and SM22α (green) expression and localization in carotid arteries of WT and *IRF9*-KO mice treated with EX527 or untreated at 14 days post injury. The proportion of PCNA-positive cells and OD values for Cyclin D1, α-SMA and SM22α were calculated (*n*=3–6 per group). Scale bar, 20 μm. (**c**) HE-stained sections show the structure of carotid arteries of NTG and SMC-*IRF9*-TG mice treated with SRT1720 or untreated at 14 days post injury. The insets in the upper panels were magnified, and the magnified images are presented in the lower panels. The black arrows indicate inner elastic discs. Scale bar, 50 μm. The intimal areas and intima/media ratios were quantified (*n*=5–11 per group). (**d**) Immunofluorescence staining shows PCNA and Cyclin D1 (red) as well as α-SMA and SM22α (green) expression and localization in carotid arteries of NTG and SMC-*IRF9*-TG mice treated with SRT1720 or untreated at 14 days post injury. The proportion of PCNA-positive cells and OD values for Cyclin D1, α-SMA and SM22α were calculated (*n*=3–6 per group). Scale bar, 20 μm. (**a**–**d**) The values are presented as the mean±s.d. *P<0.05 versus untreated WT or NTG groups, ^#^*P*<0.05 versus the EX527-treated WT groups or SRT1720-treated NTG groups, ^†^*P*<0.05 versus the untreated *IRF9*-KO or SMC-*IRF9*-TG groups. Statistical analysis was carried out using the one-way ANOVA test.

In addition to the *in vitro* experiments, we examined whether the modulation of SIRT1 activity would counteract the effect of IRF9 on neointima formation. We performed carotid artery wire injury surgery on *IRF9*-KO and WT mice; specifically, the mice were intraperitoneally injected with the SIRT1-specific inhibitor EX527 every day for 14 days, and the injured arteries were then examined on day 14. EX527 treatment enlarged the intima area (Fig. 8a), promoted PCNA and Cyclin D1 induction, and decreased α-SMA and SM22α expression (Fig. 8b) after injury in both the WT and *IRF9*-KO mice. Similarly, we treated SMC-*IRF9*-TG and NTG mice with the SIRT1-specific activator SRT1720 and examined neointima formation. Regardless of the genotype, SRT1720 treatment significantly reversed the intima area enlargement (Fig. 8c) and the gene expression alterations (Fig. 8d) caused by IRF9 overexpression after VSMC injury. Collectively, the loss- and gain-of-function studies described above indicate that IRF9-mediated neointima formation in response to vascular injury is dependent on SIRT1 deacetylase activity.

## Discussion

**Figure 9:**
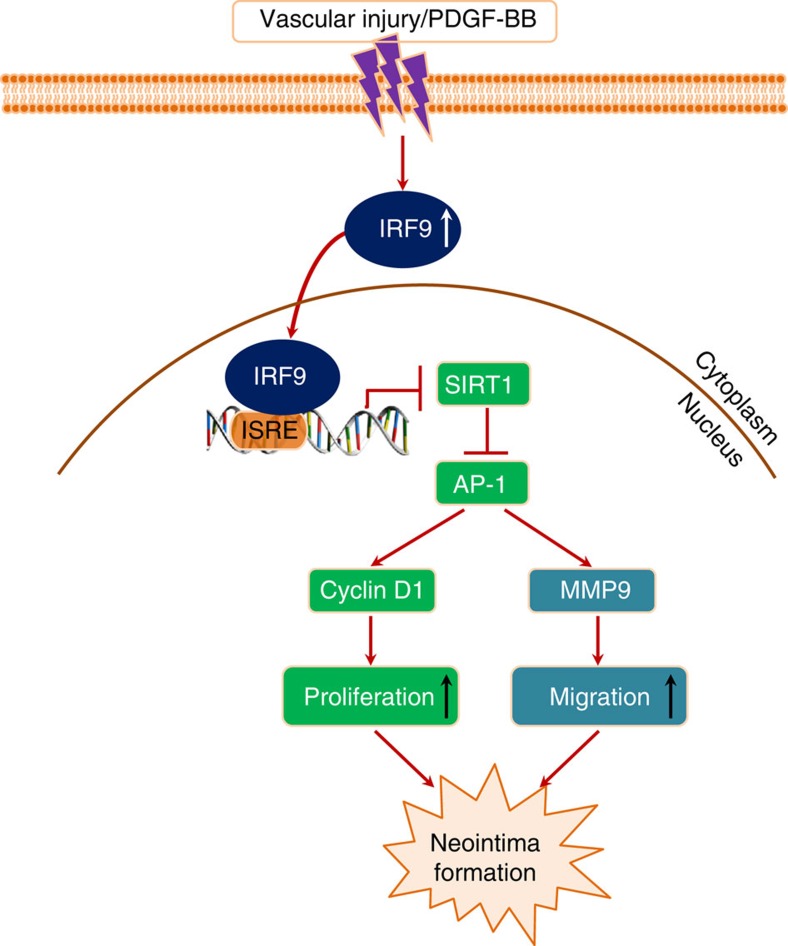
Schematic summary. Our results portray an ‘IRF9–SIRT1 axis’ in modulating neointima formation. IRF9 is induced and activated in VSMCs by arterial injury *in vivo* or PDGF-BB *in vitro*. The activated IRF9 subsequently translocates from the cytoplasm into the nucleus and suppresses SIRT1 expression (and consequently reduced SIRT1 deacetylase activity) by directly binding to an ISRE in the SIRT1 promoter. Thereby, IRF9 relieves the suppression of AP-1 transactivation by SIRT1 and promotes the expression of AP-1 downstream effectors, including Cyclin D1 and MMP9. By this means, the responsive induction and activation of IRF9 promotes VSMC proliferation, migration and neointima formation.

Neointima formation is a common pathophysiological process that occurs during several severe vascular pathologies, including atherosclerosis, in-stent restenosis and vein bypass graft failure^1^. The proliferation and migration of VSMCs upon stimulation by endogenous and exogenous insults are of prime importance in this process^21^. However, the mechanisms underlying these VSMC responses are poorly understood. In the present study, we uncovered a previously unrecognized ‘IRF9–SIRT1 axis’ in VSMCs that mediates neointima formation following vascular injury. Our results showed that IRF9 was markedly induced in VSMCs by arterial injury *in vivo* or PDGF-BB *in vitro*. The induction of IRF9 subsequently suppressed SIRT1 expression (and consequently reduced SIRT1 deacetylase activity) by directly binding to an ISRE in the SIRT1 promoter; by this means, IRF9 stimulated VSMC proliferation and neointima formation (Fig. 9). Consistent with this idea, we found that SMC-specific IRF9 overexpression worsened intimal hyperplasia, whereas *IRF9*-KO significantly alleviated injury-induced neointima formation. Importantly, genetic manipulation of SIRT1 in SMCs or pharmacological modulation of SIRT1 activity sufficiently reversed the neointima-forming effect of IRF9. On the basis of the results of the present study, we propose that the ‘IRF9–SIRT1 axis’ be considered a therapeutic target for the prevention of restenosis and warrants additional validation and investigation.

In seeking ways to effectively suppress pathological intimal thickening, tremendous efforts have been made in recent decades to elucidate the underlying mechanisms of neointima formation. Using microarrays, Zohlnhofer *et al.*^22^ comprehensively compared the gene expression profile of human in-stent restenosis specimens with the profiles of normal artery specimens, cultured human SMCs and whole blood cells from patients with in-stent restenosis. In their study, 37 of the 223 differentially expressed genes, including IRF9, were involved in the activation of IFN signalling in the neointima^22^. In agreement with their results, we found that IRF9 expression increased during neointima formation. However, unlike other IRF family members (including IRF1 and IRF7), which are highly expressed in both neointima and blood cells from patients, IRF9 is exclusively elevated in the neointima according to Zohlnhofer *et al.*^22^ The results provided by authors suggest that IRF9 involvement in neointima formation is unlikely because of its presence in haematopoietic cells and that instead IRF9 functions in VSMCs might be crucial. In the current study, we confirmed this hypothesis using immunofluorescence staining of an SMC marker, α-SMA, through which we explicitly determined that IRF9 was predominantly expressed in mouse arterial VSMCs after injury. In addition, by utilizing SMC-*IRF9*-TG mice and *IRF9*-KO mice, we found that SMC-specific IRF9 overexpression enhanced intimal hyperplasia, whereas IRF9 deficiency attenuated intimal thickening. Although the relative contribution of cells with different origins (for example, adventitial fibroblasts and haematopoietic stem cells) to neointima formation remains to be determined, our results strongly suggest that SMCs with IRF9 function are particularly important in neointima formation. To further resolve the mechanism of IRF9 function, we performed genome-wide microarray analyses using artery tissues from SMC-*IRF9*-TG and NTG mice. Notably, besides many proliferation- and migration-related genes, we found that SIRT1 and SIRT1-dependent molecular targets were also markedly altered in response to wire injury. Subsequent *in vivo* and *in vitro* experiments validated that the neointima formation mediated by IRF9 was indeed dependent on its regulation of a neointima formation modulator, SIRT1, in VSMCs.

SIRT1 is a NAD^+^-dependent class III deacetylase, which is the closest homologue of yeast Sir2 in the mammalian sirtuin family^23^. Through the deacetylation of histones and a myriad of transcription factors, SIRT1 regulates a broad range of biological processes^24^,^25^. Most studies have characterized SIRT1 as a survival factor that protects against aging and age-related diseases, including metabolic disorders, neurodegeneration, cancers and, importantly, cardiovascular diseases^26^,^27^. In addition, SIRT1 protects against vascular pathologies^20^. By deacetylating downstream targets, SIRT1 improves endothelial function, preserves blood vessel dilation, alleviates vascular inflammation and inhibits atheroma progression^28^,^29^,^30^,^31^,^32^. Notably, our previous study demonstrated that SIRT1 expression is downregulated in response to vascular injury and that SIRT1 overexpression in VSMCs prevents injury-induced neointima formation^4^. This vascular silencing effect of SIRT1 is dependent on its inhibition of genes downstream of AP-1, namely Cyclin D1 and MMP9 (ref. 4), the main functions of which are to facilitate VSMC proliferation and migration, respectively. Although the decrease in SIRT1 expression in VSMCs mediates neointima formation following vascular injury, the underlying molecular mechanism by which SIRT1 is downregulated remains unknown. In the present study, we found that IRF9 is the upstream regulator of SIRT1 in the setting of neointima formation. We demonstrated that the induction of IRF9 after vascular injury leads to the suppression of SIRT1 expression in VSMCs and subsequent intimal hyperplasia. As a prominent mediator of energy metabolism, SIRT1 expression is induced by a low-energy state through CREB, FOXOs and PPARα/β/σ^33^,^34^,^35^,^36^,^37^ and suppressed by a high-energy state through ChREBP, HIC1 and PPARγ^33^,^38^,^39^. However, little is currently known about the transcriptional regulation of SIRT1 in response to acute stresses other than energy fluctuations. Here we filled this knowledge gap by demonstrating that IRF9 negatively regulates SIRT1 expression upon arterial injury. After injury, IRF9 is elevated and binds to the ISRE in the SIRT1 promoter. Likely in cooperation with other unidentified nuclear factors, IRF9 then inhibits SIRT1 transcription in VSMCs. Furthermore, through multiple genetic and pharmacological gain- and loss-of-function studies, we clearly delineated an ‘IRF9–SIRT1 axis’ that mediates the intimal hyperplastic response to vascular injury.

In our previous study, we demonstrated that IRF9 inhibits the development of cardiac hypertrophy, and we determined that IRF9 directly interacts with myocardin to suppress its activity and the transcription of SRF (serum response factor) downstream genes in cardiomyocytes^19^. Considering that myocardin is also involved in the regulation of SMC phenotypic switching, we tested in the present study whether the downregulation of myocardin transactivation also mediates the neointima-forming effect of IRF9 in VSMCs. However, we failed to detect the interaction between IRF9 and myocardin through immunoprecipitation in cultured VSMCs. In addition, no significant change was observed in the CArG (SRF response element)-luciferase activity upon IRF9 overexpression or knockdown, either. These results demonstrated that IRF9 had no effect on myocardin activity in VSMCs *in vitro*, and whether myocardin involved in IRF9-induced neointima formation *in vivo* remains to be determined in the future study. Although the expression of SMC-selective genes was observed to be downregulated by IRF9 in the present study, this could be a secondary effect of IRF9 inhibiting the proliferation and migration of VSMCs through the SIRT1/AP-1 pathway as demonstrated here. In addition, SIRT1 was recently implicated in the preservation of VSMC differentiation^40^; therefore, the suppression of SIRT1 by IRF9 may lead to decreased expression of SMC differentiation markers, which also contribute to IRF9’s effect of promoting neointima formation.

Our finding that IRF9 mediates VSMC proliferation and intimal hyperplasia mirrors previous reports that IRF9 regulates cell survival. IRF9 was found to be overexpressed in approximately half of human breast and uterine tumour samples^13^, and IRF9 was also reported to confer resistance to antiproliferative drugs^13^,^41^. In contrast, IRF9 also possesses the pro-apoptotic function of type I IFNs by inducing p53 (ref. 12). Hence, IRF9 exhibits contradictory dual roles in the regulation of cell survival, and the role that dominates might strictly depend on the type of cell and stimuli. When stimulated by viral infection and type I IFNs, IRF9 tends to induce p53 expression and mediate pathogen-induced apoptosis^12^. However, as shown in our present work, when stimulated by vascular injury and growth factors (such as PDGF-BB), IRF9 tends to enhance VSMC proliferation and mediate neointima formation after injury. Most of the current literature describes IRF9 functions in relation to IFN-mediated immunity; thus, the immunity-independent roles of IRF9 are poorly understood. Recently, in addition to its immunoregulatory roles, our research unveiled a versatile role for IRF9 in diverse pathologies. In hepatocytes, the decrease in IRF9 upon overnutrition aggravates hepatic steatosis and insulin resistance^16^. In cardiomyocytes, a pressure overload-induced IRF9 decrease leads to the development of cardiac hypertrophy and heart failure^19^. Our finding that IRF9 suppresses SIRT1 in VSMCs to mediate sterile neointima formation undoubtedly adds important new evidence for a fundamental role of IRF9 outside IFN-mediated immunity.

In summary, we demonstrate that IRF9 is a critical mediator of neointima formation following vascular injury. Under physiological conditions, VSMCs express trace levels of IRF9, which facilitates SIRT1-mediated gene silencing. However, upon injury, increased IRF9 inhibits SIRT1 expression, which in turn promotes VSMC proliferation and vascular narrowing. Our findings shed new light on the roles of IRF9 outside immunity and implicate the newly identified ‘IRF9–SIRT1 axis’ in vasculoproliferative pathology modulation.

## Methods

### Human femoral artery samples

The human femoral artery samples were acquired from patients at the Peking Union Medical College Hospital, China. Informed consent was obtained from each participating patient. The procedures involving human tissue were approved by the Peking Union Medical College Hospital Institutional Review Board. The control samples were obtained intraoperatively from adjacent normal regions in femoral arteries from patients with lower limb arteriosclerosis obliterans undergoing bypass grafting. The in-stent restenotic arteries were collected from diseased femoral arteries removed during femoral artery bypass grafting.

### Animals and treatment

The animal study protocols were approved by the Animal Care and Use Committee of Renmin Hospital at the Wuhan University, China. The global IRF9 deficiency mice (*IRF9*-KO, C57BL/6J background) were kindly provided by Dr Tadatsugu Taniguchi (University of Tokyo, Tokyo, Japan)^16^. To generate SMC-specific *IRF9* transgenic mice (SMC-*IRF9*-TG), full-length mouse *IRF9* cDNA was ligated into a vector with a mouse *SM22a* promoter, a 5′-HA tag and an SV40 polyA signal; the construct was then microinjected into fertilized mouse embryos (C57BL/6J background). The successful insertion of the *IRF9* transgene into the mice was determined through genotyping using the PCR method and tail genomic DNA with the primers 5′-GGGTGGTGAGCCAAGCAGACTT-3′ and 5′-GGAGGGTTCCTGCTGGCAGTATTC-3′. Western blot analyses were employed to confirm IRF9 overexpression in the SMC-*IRF9*-TG mice. The SMC-specific *SIRT1* transgenic mice (SMC-*SIRT1*-TG) were previously generated^4^. The SMC-*SIRT1/IRF9*-TG (double transgenic) mice were produced by breeding SMC-*SIRT1*-TG mice with SMC-*IRF9*-TG mice. The *Sirt1*^*flox/flox*^ homozygous mice were generated by inserting a loxP-flanked neomycin cassette upstream of exon 4 and a third loxP site downstream of exon 4 in the target gene^42^. The floxed mutation does not affect SIRT1 expression in homozygous mice. *SM22-Cre* mice (B6.Cg-Tg (Tagln-cre) 1Her/J; Stock Number: 017491; Jackson Laboratory) expressing Cre recombinase were bred with *Sirt1*^*flox/flox*^ mice to generate SMC-conditional *SIRT1*-knockout mice (SMC-*SIRT1*-KO). SMC-*SIRT1*-KO mice were then mated with *IRF9*-KO mice to produce SMC-*SIRT1/IRF9*-KO (DKO) mice. The *SM22-Cre* mice were genotyped using the primers 5′-GCGGTCTGGCAGTAAAAACTATC-3′ and 5′-GTGAAACAGCATTGCTGTCACTT-3′. The primers used for *Sirt1*^*flox/flox*^ and SMC-*SIRT1*-KO mouse PCR genotyping were as follows: primer 1, 3′-GGTTGACTTAGGTCTTGTCTG-5′; primer 2, 3′-CGTCCCTTGTAATGTTTCCC-5′; and primer 3, 3′-AGGCGGATTTCTGAGTTCGA-5′. Primers 1 and 2 were used to genotype the *Sirt1*^*flox/flox*^ mice, and primers 2 and 3 were used to genotype the SMC-*SIRT1*-KO mice. Both the SMC-*SIRT1*-KO mice and DTG mice were identified through western blot analysis. Male mice (10–12 weeks old) that weighed between 25 and 30 g were used in all experiments and maintained in temperature- and humidity-controlled rooms on a 12/12-h light/dark cycle. Food and water were available *ad libitum*.

The SIRT1 inhibitor EX527 (Tocris Bioscience, Bristol, UK, 2780) was dissolved in dimethyl sulfoxide (DMSO) at a stock concentration of 50 mM that was then diluted to 0.1–1.0 nM with ultrapure water (the final DMSO concentration was <2%). The freshly prepared EX527 (2 mg kg^−1^) was injected intraperitoneally into the WT and *IRF9*-KO mice daily for 2 weeks. The NTG and SMC-*IRF9*-TG mice were treated with SRT1720 (Cat. No. S1129; Selleck, Houston, USA); the SIRT1 agonist was administered in parallel with EX527 at 100 mg kg^−1^ d^−1^ for 2 weeks. The same volume of DMSO was injected intraperitoneally into the control mice. The solutions were sterile-filtered through 0.2-μm syringe filters (Pall Corporation, MI, USA).

### Carotid artery wire injury model

For carotid artery injury mouse model, mice were anaesthetised with sodium pentobarbital (80 mg kg^−1^, intraperitoneally). A midline neck incision was made, and the left carotid artery was carefully dissected under a dissecting microscope. The external carotid artery was ligated with an 8–0 suture immediately proximal to the bifurcation point. Vascular clamps were applied to interrupt the internal and common carotid arterial blood flow. A transverse incision was made immediately proximal to the suture around the external carotid artery. A guidewire (0.38 mm in diameter, No. C-SF-15-15; Cook, Bloomington, USA) was then introduced into the arterial lumen towards the aortic arch and withdrawn five times with a rotating motion. After the guidewire was carefully removed, the vascular clamps were removed, and blood flow was restored. The skin incision was then closed. The sham littermate control mice underwent the same procedures without the incision and injury. The animal tissues were collected at specific time points after surgery for morphological and biochemical assays.

### Histological and morphometric analysis

At 0, 7, 14 or 28 days post injury, the mice were killed through an intraperitoneal injection of an overdose of sodium pentobarbital (150 mg kg^−1^). The carotid arteries were harvested after circulation perfusion and fixed with 4% paraformaldehyde dissolved in PBS. The arteries were further formalin-fixed and embedded with paraffin. Serial cross-sections (3 μm) were produced from the entire region (~300 μm) at the bifurcation site of the left carotid artery. For morphometric analysis, the sections were stained with haematoxylin and eosin after deparaffinization and rehydration. The level of neointima formation was determined based on the intima areas and I/M ratios using the Image Pro Plus software (version 6.0, Media Cybernetics) by a single observer who was blinded to the treatment protocols. A mean value was generated from five independent sections of each artery sample.

### Immunofluorescence staining

After a 5-min high-pressure antigen retrieval process (sodium citrate buffer, 100 × , pH 6.0), the arterial sections were blocked in PBS with 10% goat serum for 1 h and incubated overnight with primary antibodies at 4 °C. RASMCs were seeded on glass coverslips placed in 24-well plates. Cells were fixed with freshly prepared 4% paraformaldehyde for 15 min, followed by permeabilization with 0.2% Triton X-100 in PBS for 5 min. Next, the slides were washed in PBS containing 10% goat serum and incubated overnight with primary antibodies at 4 °C. We used antibodies against IRF9 (sc-10793; 1:100; rabbit; Santa Cruz Biotechnology), α-SMA (ab7817; 1:100; mouse; Abcam), CD31 (ab24590; 1:50; mouse; Abcam), PCNA (no. 2586; 1:100; mouse; Cell Signaling Technology), Cyclin D1 (no. 2978; 1:25; rabbit; Cell Signaling Technology), α-SMA (ab5694; 1:100; rabbit; Abcam), SM22α (ab14106; 1:100; rabbit; Abcam), smoothelin (sc-28562; 1:100; rabbit; Santa Cruz Biotechnology), OPN (BS1264; 1:100; rabbit; Bioworld) and SIRT1 (3931; 1:100; rabbit; Cell Signaling Technology). The sections were then washed with PBS and incubated with the appropriate secondary antibodies, including Alexa Fluor 488-conjugated goat anti-mouse IgG (A11001; 1:200; Invitrogen, Carlsbad, CA, USA), Alexa Fluor 488-conjugated goat anti-rabbit IgG (A11008; 1:200; Invitrogen), Alexa Fluor 568-conjugated goat anti-mouse IgG (A11004; 1:200; Invitrogen) or Alexa Fluor 568-conjugated goat anti-rabbit IgG (A11011; 1:200; Invitrogen) for 1 h at 37 °C. The nuclei were stained with 4',6-diamidino-2-phenylindole. The images were obtained using a confocal laser scanning microscope (Fluoview 1000; Olympus) or a fluorescence microscope (OLYMPUS DX51, Japan) and DP2-BSW software (version 2.2). The integrated optical density values were obtained using the Image Pro Plus software (version 6.0, Media Cybernetics).

### Cell culture and adenovirus infection

HASMCs and the mouse VSMC line MOVAS were acquired from the American Type Culture Collection (ATCC). The primary VSMCs were enzymatically isolated from the thoracic aortas of male C57BL/6J mice and Sprague–Dawley rats through enzymatic digestion. The cells were cultured in Dulbecco’s modified Eagle’s medium (DMEM)/F12 medium with 10% fetal bovine serum (FBS, SV30087.02; HyClone) and 1% penicillin-streptomycin in a 5% CO_2_/water-saturated incubator at 37 °C. The cells used in the experiments were passaged three to five times. AdshIRF9 and AdIRF9 were previously generated^19^. To knockdown SIRT1 expression, three SureSilencing mouse shSIRT1 constructs were acquired from SABiosciences (KM05054G), and the construct that most significantly reduced the SIRT1 levels was used in further experiments (designated AdshSIRT1). AdshRNA was used as a nontargeting control. The primary mouse VSMCs and RASMCs were infected with recombinant adenoviruses at a multiplicity of infection of 25 particles per cell for 24 h.

### Cell proliferation measurement using BrdU

SMC DNA synthesis was evaluated based on the level of (BrdU incorporated. Primary mouse SMCs (5 × 10^3^ per well) were growth-arrested in a 96-well microplate. After growing to 60% confluence, the cells were serum-starved for 24 h and subsequently treated with 20 ng ml^−1^ of PDGF-BB (ProSpec; Rehovot, Israel) for 48 h. BrdU was added for the last 2 h of treatment. BrdU incorporation was determined using a cell proliferation ELISA kit (Roche Diagnostics, Mannheim, Germany) in accordance with the manufacturer’s protocol.

### Western blot analysis

Cellular and mouse tissue proteins were extracted using radioimmunoprecipitation assay (RIPA) lysis buffer (720 μl of RIPA, 20 μl of phenylmethylsulphonyl fluoride, 100 μl of complete protease inhibitor cocktail (no. 4693124001, Roche), 100 μl of Phos-stop (no. 4906837001, Roche), 50 μl of NaF and 10 μl of Na_3_VO_4_ in a final volume of 1 ml). After full homogenization on ice, the samples were centrifuged; the supernatants were then separated on 10% SDS–PAGE (Invitrogen, NP0301BOX) and transferred to an Immobilon-FL membrane (Millipore, IPVH00010). After blocking with Tris-buffered saline containing 5% non-fat milk, the membranes were probed with primary antibodies against IRF9 (sc-10793; rabbit 1:200; Santa Cruz Biotechnology), PCNA (no. 2586; 1:1,000; mouse; Cell Signaling Technology), Cyclin D1 (no. 2978; 1:1,000; rabbit; Cell Signaling Technology), MMP9 (no. 2270; 1:1,000; rabbit; Cell Signaling Technology), α-SMA (ab7817; 1:1,000; mouse; Abcam), SM22α (ab14106; 1:300; rabbit; Abcam), smoothelin (sc-28562; 1:200; rabbit; Santa Cruz Biotechnology), OPN (BS1264; 1:500; rabbit; Bioworld), SIRT1(sc-15404; 1:200; rabbit; Santa Cruz Biotechnology) and GAPDH (MB001; 1:10,000; mouse; Bioworld) overnight at 4 °C. To examine the acetylation status of AP-1, the cellular and mouse tissue protein lysates were immunoprecipitated using total c-Jun (no. 9165; 1:50; rabbit; Cell Signaling Technology)^31^. The immunoprecipitates were immunoblotted using an acetylated-lysine antibody (no. 9441; 1:1,000; rabbit; Cell Signaling Technology). The membrane was then incubated with a secondary IRDye 800CW-conjugated antibody (Li-Cor Biosciences, at 1:10,000 dilution, Lincoln, NE, USA), and the Odyssey Imaging System (Li-Cor Biosciences) was used for signal detection. Protein expression levels were quantified and normalized to the GAPDH loading control. Uncropped scanned images of all western blots presented in this manuscript are available as Supplementary Fig. 10.

### Migration assay

SMC migration assay was determined using a modified Boyden chamber^43^. In brief, murine aortic SMCs were trypsinized and washed. Once resuspended, ~5 × 10^4^ cells were added to the top wells of transwell-modified Boyden chambers of a 24-well transwell dish (a 6.5-mm polycarbonate membrane containing 8-mm pores; Corning, NY, USA) and allowed to attach for 30 min. SMCs were exposed to medium with or without PDGF-BB (20 ng ml^−1^) that was added to the lower chamber for 6 h. The cells that migrated to the bottom of membranes were fixed and stained with 0.1% crystal violet/20% methanol and counted. Five randomly chosen high-power fields ( × 200) in three independent experiments were used to calculate the average number of migrated cells. Images were quantified using the Image Pro Plus software.

### Gelatin zymography

Mouse carotid arteries were harvested from the sham operation and 28-day post-injury groups and the proteins were extracted with lysis buffer (50 mM Tris-HCl, pH 6.8, 10% glycerol and 1% SDS). Equal amounts of tissue extract protein (15 μg) were loaded on 10% SDS–PAGE gels containing 1% gelatin to detect Gelatinase activity. After washing in 2.5% Triton X-100, the gels were incubated overnight in buffer (10 mM CaCl_2_, 0.01% NaN_3_ and 50 mM Tris-HCl, pH 7.5). Subsequently, the obtained gels were stained with 0.2% Coomassie blue R-250 (Bio-Rad) for 2 h and then destained with 10% acetic acid and 40% methanol. Signals were detected using a Nikon D700 digital camera.

### SIRT1 activity measurement

SIRT1 activity was determined using a deacetylase SIRT1 Fluorometric Kit (Biomol International) according to the manufacturer’s instructions. Briefly, SIRT1 was immunoprecipitated using a SIRT1 antibody (Santa Cruz Biotechnology) from the carotid artery and primary mouse VSMC homogenates (200 μg of protein) in RIPA buffer. The SIRT1 substrate reagent and NAD^+^ were added to the SIRT1-conjugated beads and incubated at 37 °C for 80 min after a final washing. The substrate–SIRT1 mixture was placed on a 96-well plate, and the developer reagent was added to the wells at 37 °C for 20 min. The plate was read using a spectrophotometer with an excitation of 405 nm.

### Luciferase reporter assays

The Myc–IRF9 constructs were generated by amplifying the *Irf9* gene-encoding region with the primers 5′-CCGGAATTCATGGCATCAGGCAGGGCACG-3′ and 5′-CCGCTCGAGCTACACCAGGGACAGAATGGCTG-3′ from HA-IRF9 and subcloning the amplicon into Myc-C1 to generate Myc-IRF9. The SIRT1-luc plasmid was generated by cloning ~1,470 bp of the *Sirt1* promoter (−1,490 to −20) into the **pGL3-basic** vector (Promega, Madison) using the primers 5′-CCGCTCGAGCCCTCCTCCCTCGCCTCCT-3′ (forward) and 5′-CCGACGCGTCCTAATGCTCTCCCTCCCCT-3′ (reverse). The mouse SIRT1 promoter (NC_000076.6, 63340636- 63339293, negative strand) was amplified from the C57BL/6 genome using pGL3-SIRT1P-F/R primers. The PCR primers are presented in Supplementary Table 2. The PCR product was purified, enzyme-digested and inserted into the **pGL3-basic plasmid** between the XhoI and HindIII sites. The IRF9-binding site of the SIRT1 promoter was predicted by Genomatix ( http://www.genomatix.de/) Matinspector online software. The ISRE core sequence in SIRT1 promoter was deleted using SIRT1-IRF-MF/R primers by the fusion-PCR approach described earlier (SIRT1-IRF-M1: 5′-TTTTAAATTTCGTTTTTGAGA-3′). The mouse VSMC line MOVAS was transfected with 2 μg of pGL3-SIRT1 and various amounts of Myc-empty or IRF9-Myc plasmids. The mouse primary VSMCs were treated with PDGF-BB (20 ng ml^−1^). The cells were harvested 24 h later and lysed with 100 μl of passive lysis buffer (Promega). After removing the cell debris via centrifugation, the supernatant was used for luciferase assays, which were performed using a Single-Mode SpectraMax Microplate Reader in accordance with the manufacturer’s instructions.

### Chromatin immunoprecipitation

For the ChIP assay, MOVAS cells were transfected with a Myc-tagged IRF9 plasmid or the control vector **pcDNA3.1**. After 48 h, the cells were harvested, fixed with 1% formaldehyde for 10 min and washed with ice-cold PBS. The cells were then sonicated to generate DNA fragments, which were immunoprecipitated using anti-Myc (Roche, 11814150001) and normal rabbit IgG (CST; 2729S) followed by protein G-magnetic beads (Invitrogen; 10004D). The precipitated DNA was recovered via phenol/chloroform extraction and amplified through RT–PCR (PCR with reverse transcription) using specific primers for *Sirt1*: primer 1, 5′-CATTCTGCCTCCTGAGTGCTAA-3′ (forward) and 5′-GTGGCTCACAACCACCTGTAAC-3′ (reverse); primer 2, 5′-GGCTTGCTGGTGTACATCTT-3′ (forward) and 5′-TGGTAAACACAGACATGTATGG-3′ (reverse); primer 3, 5′-AAACCTGACTAGCTGATTTCTC-3′ (forward) and 5′-GTAAGTTGAAGGTAATCTCCAA-3′ (reverse); primer 4, 5′-TCCTCAATCGCCAGATCTTTC-3′ (forward) and 5′-TTCCTGAGGAGAACTCCTCCA-3′ (reverse); and primer 5, 5′-AGGTGGCGCTCGCCCTTCA-3′ (forward) and 5′-CCACTGCTGCGCTCGGCTC-3′ (reverse).

### RNA isolation and quantitative real-time PCR

Total RNA from mouse carotid artery tissue was isolated using TRIzol reagent (Roche, 11667165001). The cDNA synthesis reaction was performed using 2 μg of total RNA and a Transcriptor First Stand cDNA Synthesis Kit (Roche, 04897030001). The quantitative real-time PCR reactions were performed in 20-μl volumes (LightCycler 480 SYBR Green I Master Mix, 04887352001, Roche) using the LightCycler 480 Real-time PCR system (Roche) in accordance with the manufacturer’s instructions. The samples were quantified by normalizing the gene expression level to that of the standard housekeeping β-actin gene and expressed as relative mRNA levels compared with the internal control. To confirm the microarray results, 36 proliferation- and migration-related genes were selected from Supplementary Table 1, and the RNAs isolated from the carotid arteries of NTG and SMC-*IRF9*-TG mice at 14 day post injury were used for real-time PCR. The real-time PCR primers were shown in Supplementary Table 3.

### Statistical analysis

The data were analysed using the SPSS software, version 13.0 (SPSS Inc., Chicago, IL, USA), and are presented as the mean values±s.d. Comparisons between the two groups were performed using independent sample *t*-test. Differences between multiple groups were determined using the analysis of a one-way analysis of variance with least significant difference or Tamhane’s T2 *post-hoc* test. *P*<0.05 was considered statistically significant.

## Author contributions

In this study, S.-M. Z., L.-H. Z. and H.-Z. C. designed and performed the experiments, analysed the data and wrote the manuscript; R.Z. and P.Z. wrote the manuscript; D.-S.J., L.G., S.T., Y.Z. and X.-F.Z. performed the experiments; P.-X.W. analysed the data; X.-D.Z. and D.-P.L. provided useful advice and helped to revise the manuscript; and H.L. (corresponding author) designed the experiments and wrote the manuscript.

## Additional information

**Accession codes:** Microarray data have been deposited in the NIH GEO repository under accession code GSE61046.

**How to cite this article:** Zhang, S.-M. *et al.* Interferon regulatory factor 9 is critical for neointima formation following vascular injury. *Nat. Commun.* 5:5160 doi: 10.1038/ncomms6160 (2014).

## Supplementary Material

Supplementary Information Supplementary Figures 1-10 and Supplementary Tables 1-3.
