# Supplementary material for: Interferon regulatory factor 9 is critical for neointima formation following vascular injury
Source: Nat Commun. 2014 Oct 16;5:5160. doi: 10.1038/ncomms6160 (PMC4218966; doi:10.1038/ncomms6160)
Supplement: Supplementary Information — Supplementary Figures 1-10 and Supplementary Tables 1-3. [file ncomms6160-s1.pdf]

Supplementary Information

Interferon Regulatory Factor 9 is Critical for Neointima Formation Following Vascular Injury

Shu-Min Zhang[1,2*], Li-Hua Zhu[1,2*], Hou-Zao Chen[3*], Ran Zhang[3], Peng Zhang[1,2], Ding-Sheng Jiang[1,2], Lu Gao[4], Song Tian[2], Lang Wang[1,2], Yan Zhang[1,2], Pi-Xiao Wang[1,2], Xiao-Fei Zhang[5], Xiao-Dong Zhang[5], De-Pei Liu[3#], Hongliang Li[1,2 #]

[1]Department of Cardiology, Renmin Hospital of Wuhan University, Wuhan 430060, China; [2] Cardiovascular Research Institute, Wuhan University, Wuhan 430060, China; [3]State Key Laboratory of Medical Molecular Biology, Department of Biochemistry and Molecular Biology, Institute of Basic Medical Sciences, Chinese Academy of Medical Sciences & Peking Union Medical College, Beijing 100005, China; [4]Department of Cardiology, Institute of Cardiovascular Disease, Union Hospital, Tongji Medical College, Huazhong University of Science and Technology, Wuhan, 430022, China.[5]College of Life Sciences, Wuhan University, Wuhan 430072, China;

Correspondence and requests for materials should be addressed to H.L.L

(E-mail: lihl@whu.edu.cn**)**

Supplementary Figures 1-10

Supplementary Table 1-3

Supplementary Figures

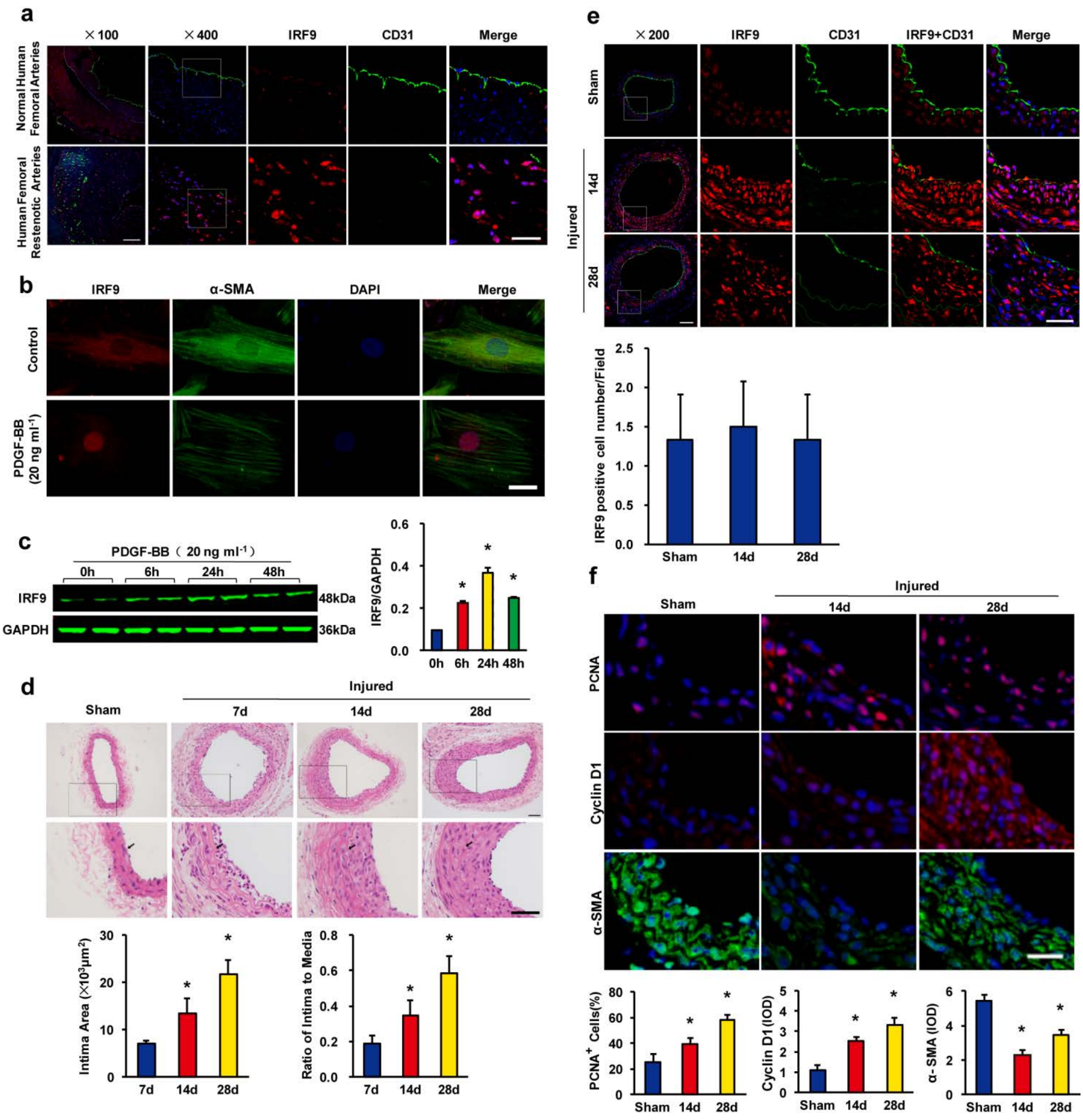

Supplementary Fig. 1 IRF9 is involved in neointima formation. (**a**) Immunofluorescence staining for IRF9 in a normal femoral artery (upper panels) and the neointima in an in-stent restenotic human femoral artery (lower panels). IRF9 is depicted in red, CD31 are shown in green, and DAPI is presented in blue. Scale bar, 50 μm. (n = 3 per group). (**b**) IRF9 (red) and α-SMA (green) immunofluorescence staining in rat aortic smooth muscle cells (RASMCs) treated with platelet-derived growth factor-BB (PDGF-BB) (20 ng ml^{-1}) for 6 hours. DAPI is shown in blue.

Scale bar, 20 μm. (n = 4 per group). (**c**) IRF9 protein levels in RASMCs were detected using western blot analyses before and at 6, 24, and 48 hours after PDGF-BB was administered (left). Right panels: quantification of IRF9 levels (n = 4, $*P < 0.05$ versus 0 h treatment). (**d**) Hematoxylin and eosin (HE)-stained sections show the arterial structures from wild-type (WT) mice that underwent a sham operation or wire injury surgery (7, 14, or 28 days after surgery). The insets in the upper panels were magnified and are presented in the lower row. The black arrows indicate inner elastic discs. Scale bar, 50 μm. The intima/media ratios and intima areas were quantified (n = 10 at 7 days, n = 15 at 14 days, n = 13 at 28 days, $*P < 0.05$ versus 7 days post-injury). (**e**) Immunofluorescence staining shows IRF9 (red) expression and localization in the arteries at various time points post-injury. The endothelial cells are indicated by CD31 (green). Scale bar, 50 μm. (n = 3 per group at each time point). (**f**) Immunofluorescence staining shows PCNA (red), Cyclin D1 (red), and α-SMA (green) expression in each row. Scale bar, 20 μm. The proportion of PCNA-positive VSMCs and the Cyclin D1 and α-SMA OD values for the arteries were calculated (n = 4-5 for sham group, n = 5-7 for 14 days-injured group, n = 6-7 for 28 days-injured group, $*P < 0.05$ versus sham group). (**c**-**f**) The values are presented as the mean ± s.d. Statistical analysis was carried out using one-way ANOVA test.

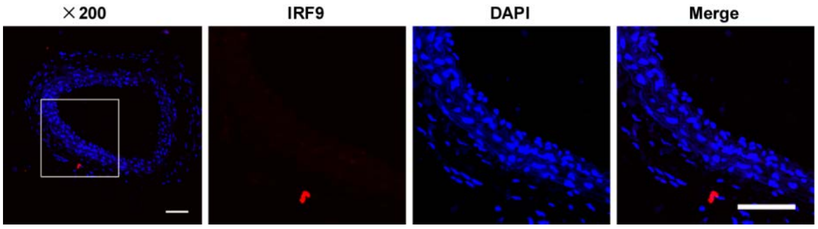

Supplementary Fig. 2 IRF9 is absent in *IRF9*-KO arteries. IRF9 immunofluorescence staining in the artery of *IRF9*-KO mice. DAPI is shown in blue, and IRF9 (if any) is displayed in red. Scale bar, 50 μm. n = 3.

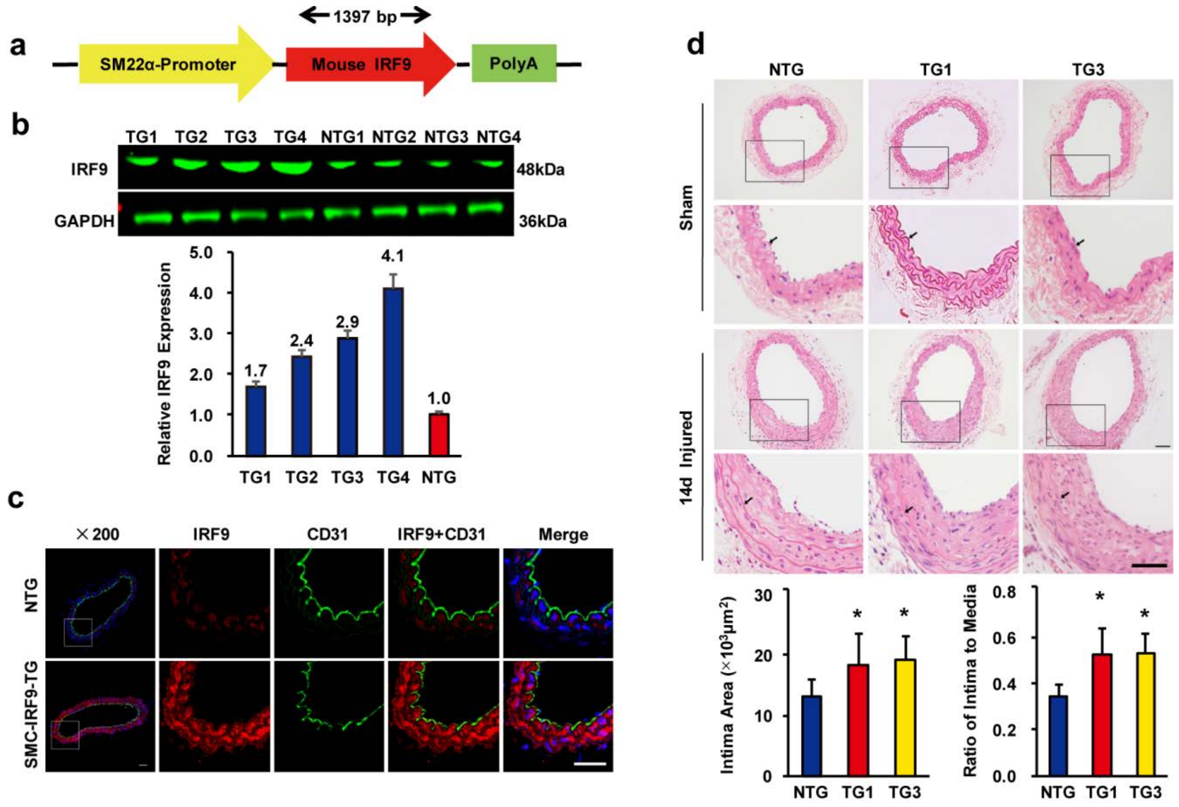

Supplementary Fig. 3 SMC-specific IRF9 overexpression promotes neointima formation. (**a**) Schematic for the SMC-specific transgenic (TG) vector structure. The vector was first constructed using a mouse *SM22α* promoter, and an SV40 polyA signal. The full-length mouse IRF9 cDNA was cloned into the vector between the *EcoRI* sites. (**b**) The arterial IRF9 protein levels in the SMC-*IRF9*-TG and non-transgenic (NTG) mice were measured by western blot analysis (n = 4 samples, 3 cartid arteries were collected as a sample). The blots represent three independent experiments. The changing folds between IRF9 expression in different SMC-*IRF9*-TG lines and the mean level of IRF9 expression in NTG mice are marked above the corresponding bars. (**c**) Immunofluorescence staining shows IRF9 (red) expression and localization in the arteries at 14 days post-injury. The endothelial cells were stained with CD31 (green). DAPI is shown in blue. Scale bar, 50 μm. (n = 3 per group). (**d**) The HE-stained sections demonstrate the NTG and SMC-*IRF9*-TG line 1 and line 3 carotid artery structures at 14 days after the wire injury surgery. Insets in the upper panels were magnified and are presented in the lower panels. The black arrows indicate inner elastic discs. Scale bar, 50 μm. The intimal areas and intima/media ratios were quantified (n = 6-14 for

sham group, n = 6-7 for 14 days-injured group, *$P < 0.05$ versus NTG group). (**b**) and (**d**) The values are presented as the mean ± s.d. Statistical analysis was carried out using one-way ANOVA test.

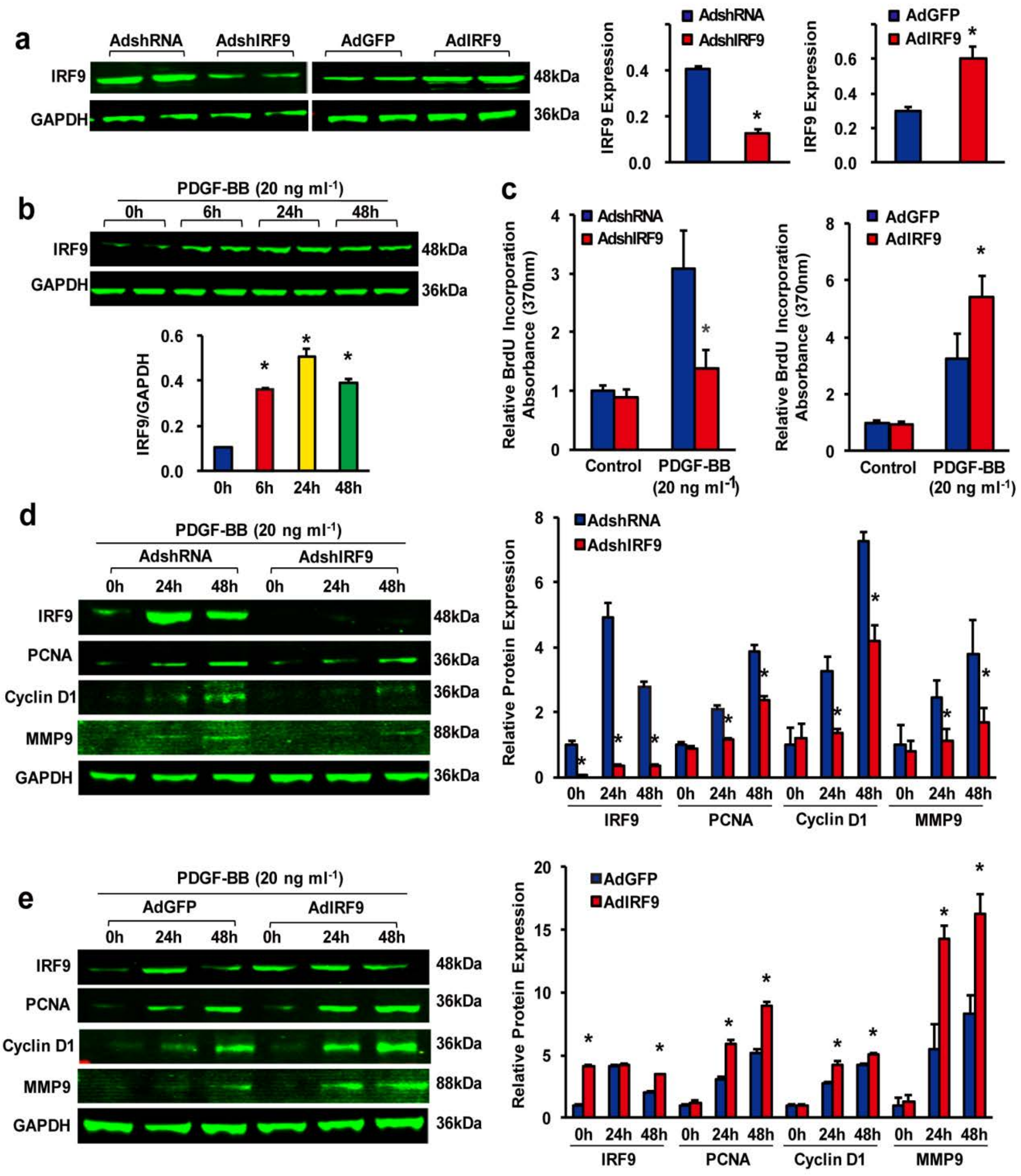

Supplementary Fig. 4 IRF9 promotes VSMC proliferation and migration in human aortic smooth muscle cells. (**a**) Immunblots verified the knockdown or overexpression of IRF9 in human aortic smooth muscle cells (HASMCs, left) and quantification of IRF9 levels (right, *$P < 0.05$ versus AdshRNA or AdGFP). (**b**) HASMCs were treated with PDGF-BB for 6, 24 and 48 hours then subjected to western blot to examine IRF9 expression. (*$P < 0.05$ versus 0 h treatment group). (**c**) The HASMCs were infected with AdshRNA and AdshIRF9 adenoviruses as well as AdGFP and AdIRF9 adenoviruses 24 hours prior to PDGF-BB administration. The cells were harvested before or at 12, 24, or 48 hours after PDGF-BB administration. BrdU incorporation was determined by

measuring the absorption at 370 nm to assess SMC proliferation upon PDGF-BB stimulation. (**P* < 0.05 versus AdshRNA or AdGFP group). The results represent three independent experiments. (**d**) **and** (**e**) IRF9, PCNA, Cyclin D1, and MMP9 protein levels in the AdshRNA and AdshIRF9 infected HVSMCs (**d**) and in the AdGFP and AdIRF9 infected HASMCs (**e**) were analyzed using western blots at various time points after PDGF-BB administration. (**P* < 0.05 versus AdshRNA or AdGFP group) (**a**)**,** (**b**)**,** (**d**) **and** (**e**) The corresponding protein levels were normalized to GAPDH and quantified. The blots are presented as representative blots from three independent experiments. (**a**-**e**) The values are presented as the mean ± s.d. Statistical analysis was carried out using independent sample *t*-test.

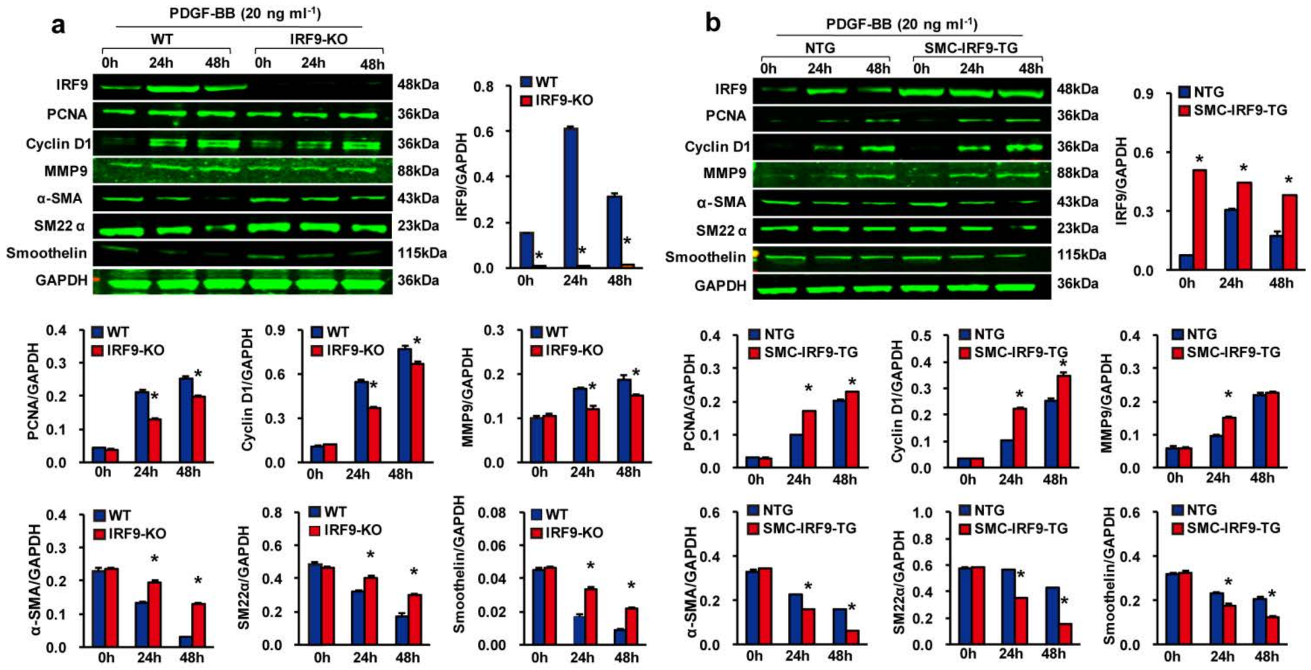

Supplementary Fig. 5 IRF9 promotes VSMC proliferation, migration and phenotypic switching in primary mouse VSMCs. (**a**) **and** (**b**) IRF9, PCNA, Cyclin D1, MMP9, α-SMA, SM22α, and Smoothelin protein levels in the *IRF9*-KO and WT primary mouse VSMCs (**a**) and in the SMC-*IRF9*-TG and NTG primary mouse VSMCs (**b**) were analyzed using western blots at various time points after PDGF-BB administration (n = 3, *$P < 0.05$ versus WT or NTG group). The corresponding protein levels were normalized to GAPDH and quantified. Blots are representative of three independent experiments. The values are presented as the mean ± s.d. Statistical analysis was carried out using independent sample *t*-test.

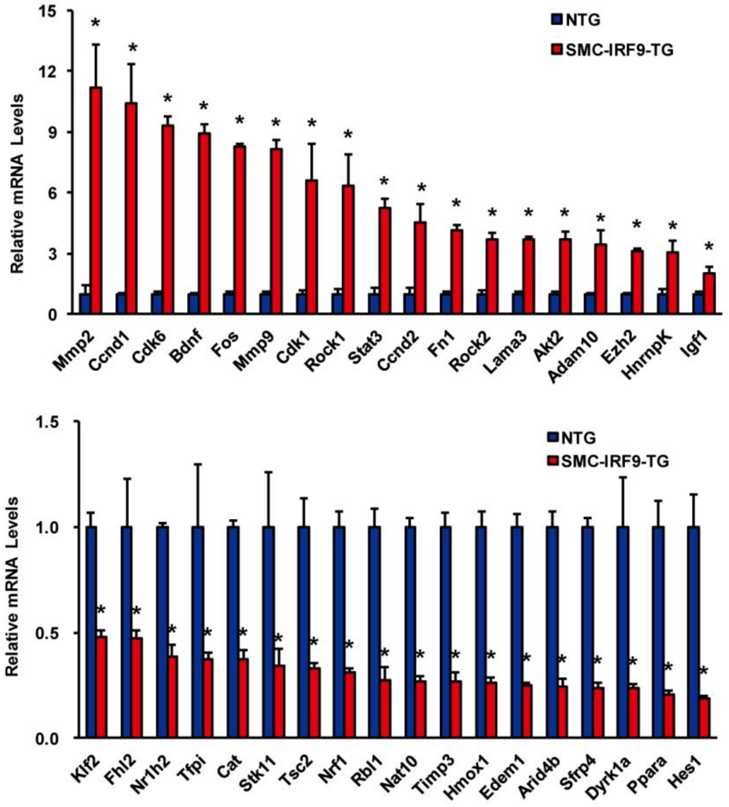

Supplementary Fig. 6 IRF9 regulates SIRT1-related genes. Real-time PCR confirms the expression of upregulated (upper panel) and downregulated (lower panel) SIRT1-related genes in the microarray results in the carotid arteries of NTG and SMC-*IRF9*-TG mice at 14 days post-injury (n = 6, *$P < 0.05$ versus NTG group). The values are presented as the mean ± s.d. Statistical analysis was carried out using independent sample *t*-test.

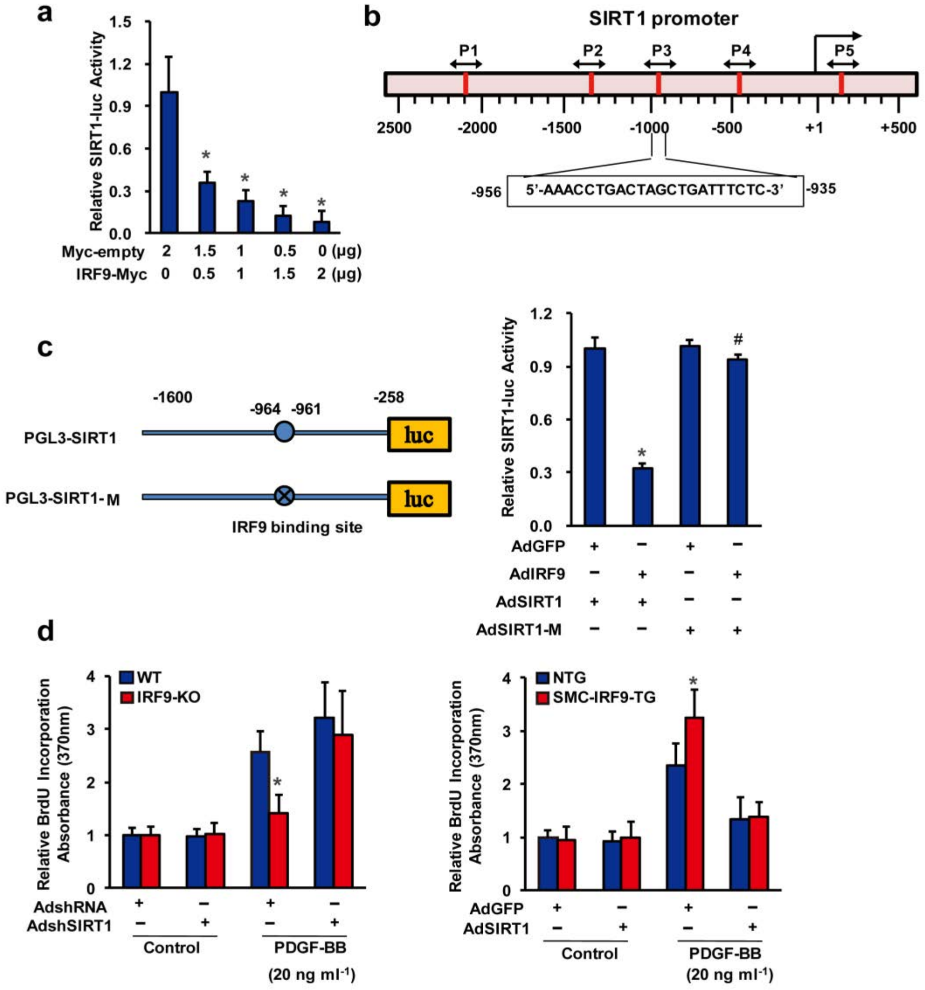

Supplementary Fig. 7 IRF9 suppresses SIRT1 transcription. (**a**) Adenovirus constructs containing SIRT1-luciferase were used to infect MOVAS cells and investigate IRF9-dependent SIRT1 expression. Two micrograms of various proportions of Myc-empty or IRF9-Myc plasmids was used to transfect MOVAS cells. After the Myc-empty or IRF9-Myc plasmid was introduced into MOVAS cells, the luciferase activity was measured. (n = 9 per group). *P < 0.05 versus Myc-empty transfected group. (**b**) Schematic diagram of five pairs of primers (P1-P5) targeted against the five putative IFN-stimulated response elements (ISREs) in the human SIRT1 promoter. (**c**) Left panel: schematic diagram of SIRT1-luc constructs with or without the IRF9 binding site in the mutated SIRT1 promoter. Right panel: relative luciferase activities of SIRT1-luc and SIRT1-mutant-luc (PGL3-SIRT1-M) upon AdIRF9 or AdGFP infection. (n = 9 per group). *P < 0.05 versus AdGFP/AdSIRT1 group, # P < 0.05 versus AdIRF9/AdSIRT1 group. (**d**) BrdU labeling was used to evaluate cell proliferation. AdshRNA- and AdshSIRT1-infected WT and *IRF9*-KO primary mouse

VSMCs (left panel) as well as AdGFP- and AdSIRT1-infected SMC-*IRF9*-TG and NTG primary mouse VSMCs (right panel) treated with 20 ng ml^{-1} PDGF-BB for 24 hours were examined along with untreated cells. (n = 9 per group). **P* < 0.05 versus WT VSMCs infected with AdshRNA or NTG VSMCs infected with AdGFP. (**a**), (**c**) and (**d**) The values are presented as the mean ± s.d. Statistical analysis was carried out using independent sample *t*-test.

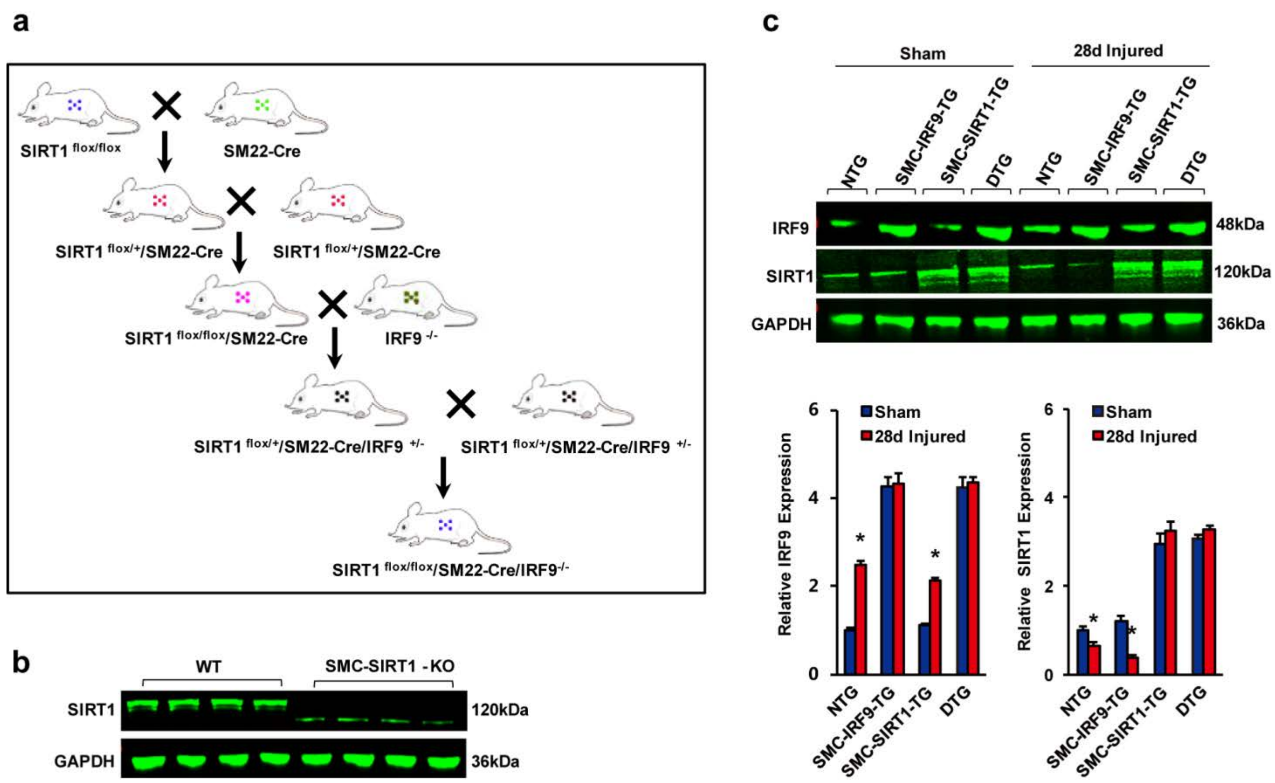

Supplementary Fig. 8 The effect of IRF9 on neointima formation is mediated by SIRT1. (**a**) Schematic diagram of the mice used to generate the SMC-specific *SIRT1* and *IRF9* double KO (DKO) mice. (**b**) The SIRT1 protein levels was examined in the corresponding mice arteries using western blots (n = 4 samples, 3-4 carotid arteries were collected as one sample). The blots represent three independent experiments. **(c)** IRF9 and SIRT1 expression in sham-operated and 28-day injured mouse arteries was examined using western blot (n = 3 samples per group at each time point, 3-4 carotid arteries were collected as one sample). The blots represent three independent experiments. The corresponding protein levels were normalized to GAPDH and quantified. $*P < 0.05$ versus the sham-operated group. The values are presented as the mean ± s.d. Statistical analysis was carried out using independent sample *t*-test.

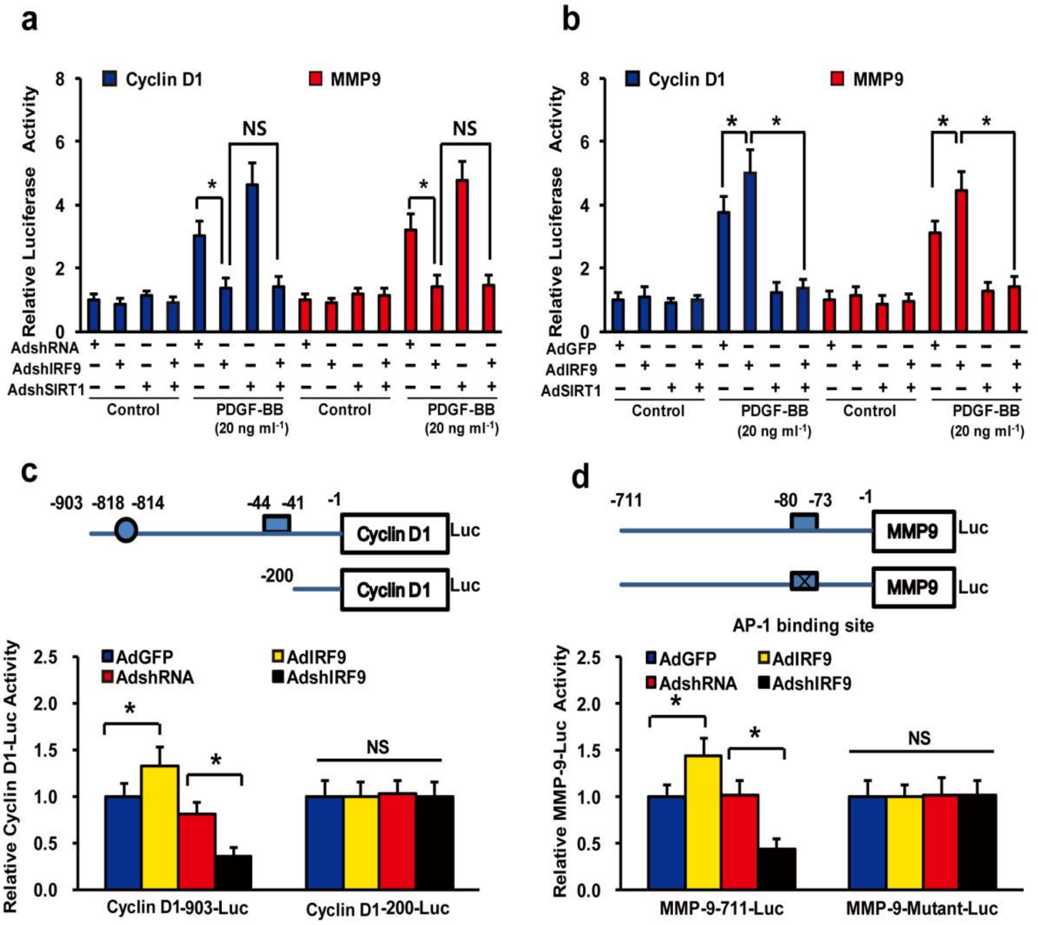

Supplementary Fig. 9 IRF9 relieves the suppression of AP-1 transactivation. (**a**) **and** (**b**) Cyclin D1 (promoter)-luc (**a**) and MMP9 (promoter)-luc (**b**) were used to examine AP-1 transactivation. To manipulate IRF9 and SIRT1 levels, AdshIRF9, AdshSIRT1, AdIRF9, and AdSIRT1 were used to infect RASMCs. PDGF-BB was also used. (**c**) **and** (**d**) Upper panels: schematic diagrams of luciferase driven by promoters of Cyclin D1 (**c**) and MMP9 (**d**) with normal or mutated/deleted AP-1 binding sites. Lower panel: corresponding luciferase activities upon manipulations of IRF9 and SIRT1 expressions. (**a-d**), the values are presented as the mean ± s.d, the results represent three independent experiments, and statistical significance is indicated: *$P < 0.05$; NS (not significant): $P \geq 0.05$. Statistical analysis was carried out using the one-way ANOVA test (**a-b**) or independent sample *t*-test (**c-d**).

Supplementary Fig. 10 Full gel scans relating to indicated figures.

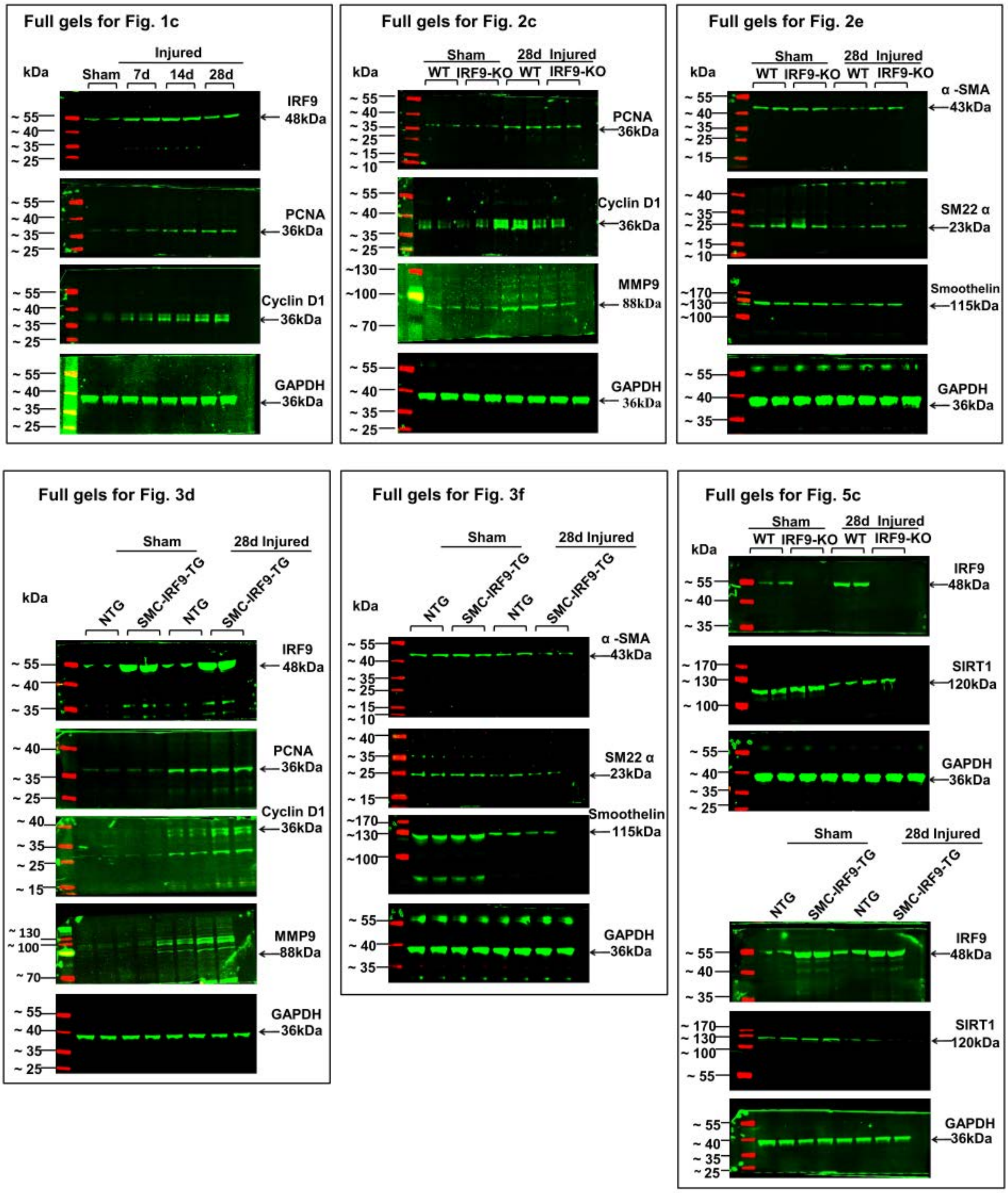

Full gels for Fig. 7e

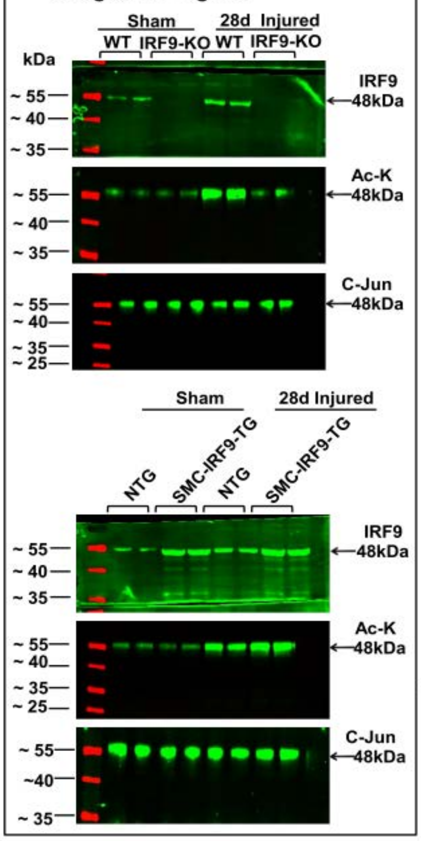

Full gels for Fig. 7f

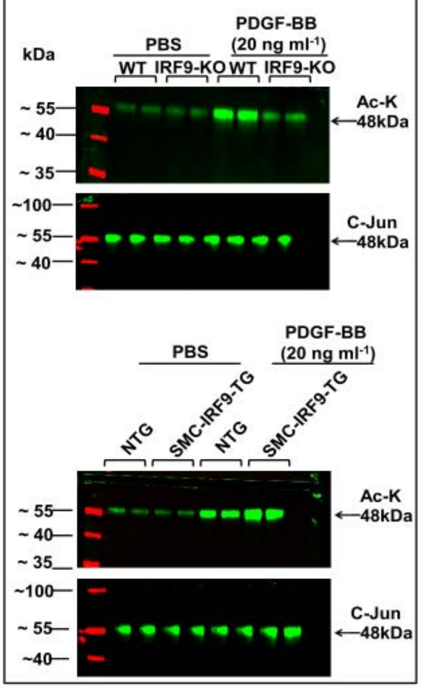

Full gels for Supplementary Fig. 1c

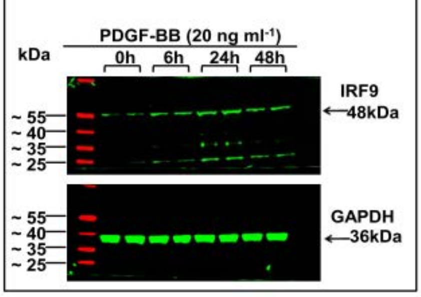

Full gels for Supplementary Fig. 3b

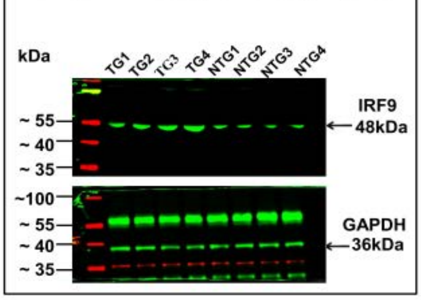

Full gels for Supplementary Fig. 4a

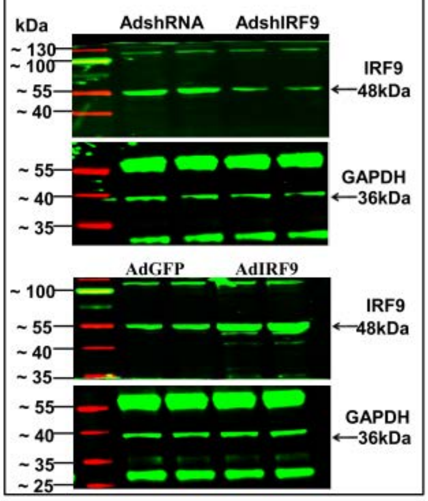

Full gels for Supplementary Fig. 4b

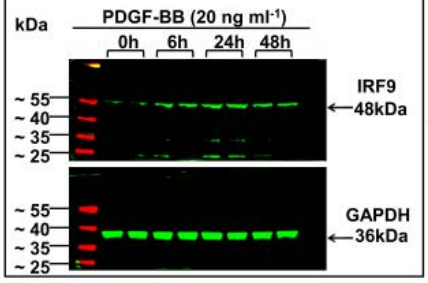

Full gels for Supplementary Fig. 4d

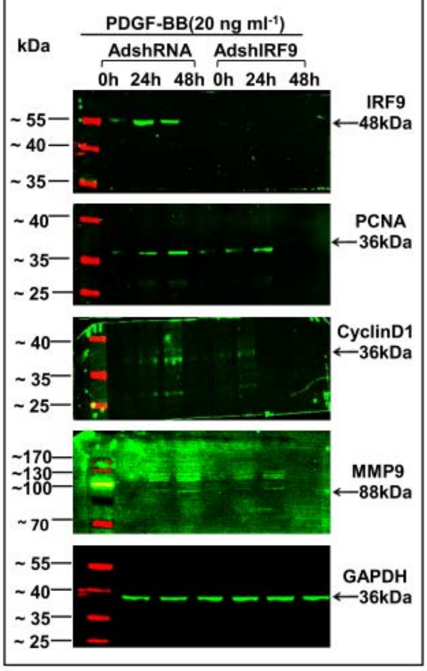

Full gels for Supplementary Fig. 4e

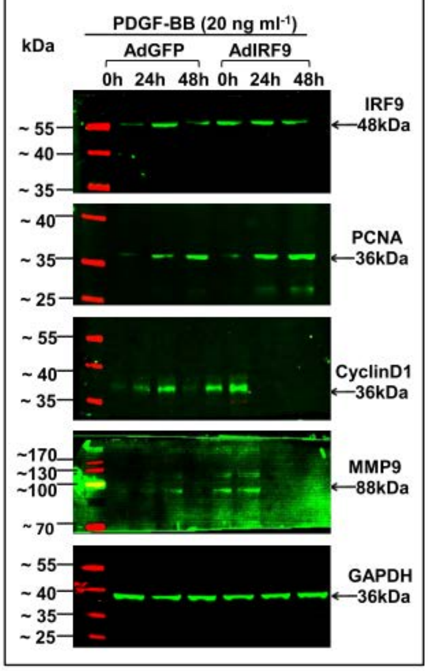

Full gels for Supplementary Fig. 5a

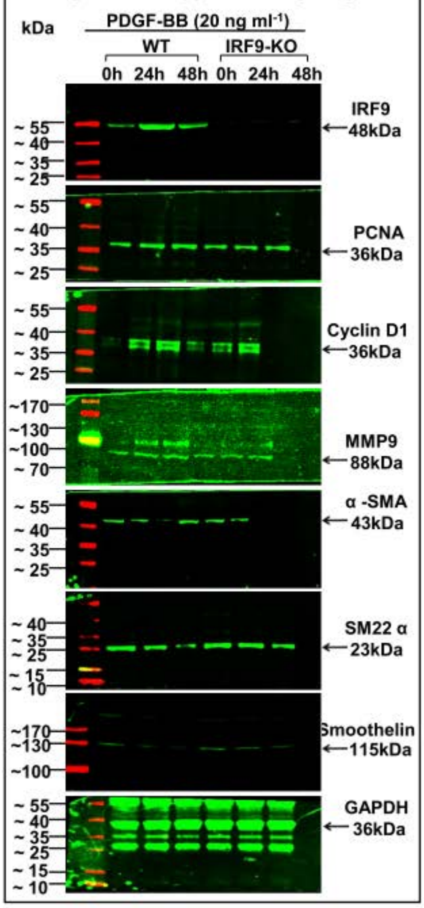

Full gels for Supplementary Fig. 5b

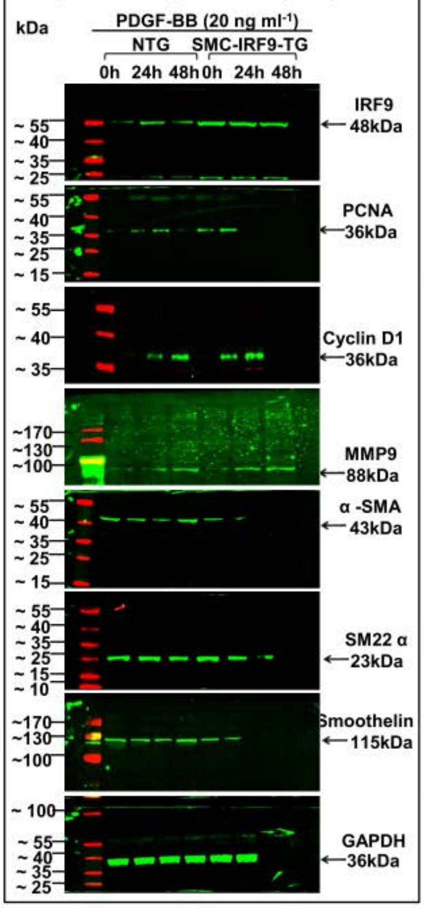

Full gels for Supplementary Fig. 8b

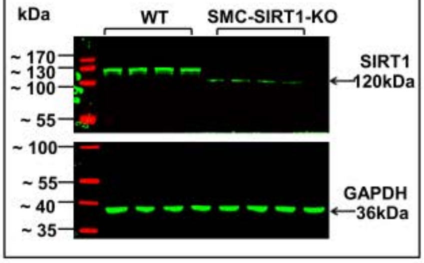

Full gels for Supplementary Fig. 8c

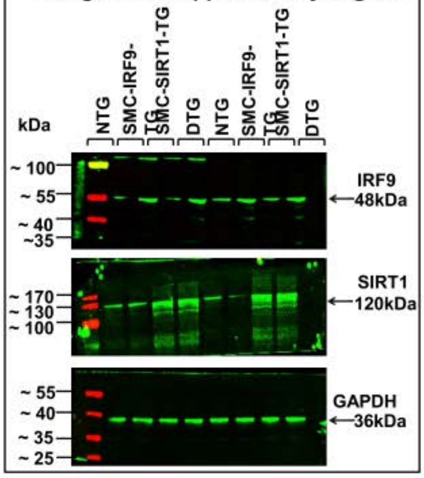

Full gels for Fig. 4d

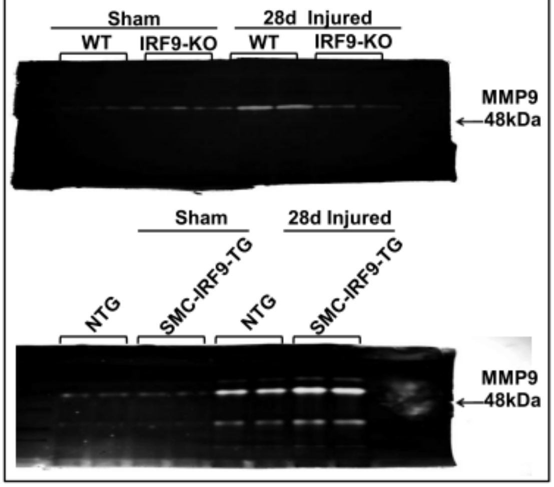

Supplementary Tables

Supplementary Table 1. SIRT1-related genes

Probeset ID	Sirt1-Related	Gene Title	P-value	Fold-Change
1439364_a_at	Mmp2	matrix metallopeptidase 2	6.96E-04	10.04
1423100_at	Fos	Fos oncogene	5.42E-04	9.25
1416122_at	Ccnd1	cyclin D1	1.90E-04	8.15
1435338_at	Cdk6	cyclin-dependent kinase 6	7.22E-03	7.99
1417947_at	Pcna	proliferating cell nuclear antigen	6.17E-03	7.96
1448314_at	Cdk1	cyclin-dependent kinase 1	7.20E-03	7.49
1448291_at	Mmp9	matrix metallopeptidase 9	3.86E-03	7.28
1437218_at	Fn1	fibronectin 1	2.68E-03	7.20
1430127_a_at	Ccnd2	cyclin D2	5.84E-03	6.90
1422168_a_at	Bdnf	brain derived neurotrophic factor	5.21E-03	6.62
1423445_at	Rock1	Rho-associated coiled-coil containing protein kinase	4.93E-04	6.52
1448694_at	Jun	Jun oncogene	4.20E-03	5.48
1449399_a_at	Il1b	interleukin 1 beta	5.79E-03	4.45
1440265_at	Jund	Jun proto-oncogene related gene d	6.95E-03	4.33
1426642_at	Fn1	fibronectin 1	5.78E-03	4.24
1421858_at	Adam17	a disintegrin and metallopeptidase domain 17	1.09E-02	4.14
1448176_a_at	Hnrnpk	heterogeneous nuclear ribonucleoprotein K	9.11E-04	4.06
1450008_a_at	Ctnnb1	catenin (cadherin associated protein), beta 1	2.04E-02	3.72
1428103_at	Adam10	a disintegrin and metallopeptidase domain 10	2.09E-02	3.54
1423444_at	Rock2	Rho-associated coiled-coil containing protein kinase	1.64E-02	3.49
1433803_at	Jak1	Janus kinase 1	1.14E-03	3.15
1424480_s_at	Akt2	thymoma viral proto-oncogene 2	4.37E-03	3.11
1424942_a_at	Myc	myelocytomatosis oncogene	8.95E-03	3.07
1419513_a_at	Ect2	ect2 oncogene	4.46E-04	2.96
1449244_at	Cdh2	cadherin 2	2.33E-04	2.95
1425895_a_at	Id1	inhibitor of DNA binding 1	3.97E-03	2.84
1427512_a_at	Lama3	laminin, alpha 3	9.14E-03	2.65
1425514_at	Pik3r1	phosphatidylinositol 3-kinase, regulatory subunit, po	1.43E-02	2.63
1420380_at	Ccl2	chemokine (C-C motif) ligand 2	7.31E-04	2.51
1421315_s_at	Cttn	cortactin	2.45E-02	2.39
1430533_a_at	Ctnnb1	catenin (cadherin associated protein), beta 1	2.19E-02	2.30
1460700_at	Stat3	signal transducer and activator of transcription 3	2.30E-02	2.22
1425515_at	Pik3r1	phosphatidylinositol 3-kinase, regulatory subunit, po	1.35E-03	2.07
1422503_s_at	Parp1	poly (ADP-ribose) polymerase family, member 1	7.89E-03	2.06
1450297_at	Il6	interleukin 6	1.15E-03	2.05
1422169_a_at	Bdnf	brain derived neurotrophic factor	2.33E-03	2.04
1452014_a_at	Igf1	insulin-like growth factor 1	2.56E-02	1.85
1442884_at	Hgf	hepatocyte growth factor	1.74E-03	1.82
1416544_at	Ezh2	enhancer of zeste homolog 2 (Drosophila)	2.48E-02	1.77
1417065_at	Egr1	early growth response 1	1.87E-04	1.75
1416353_at	Nr1h2	nuclear receptor subfamily 1, group H, member 2	7.49E-03	-1.51
1418644_a_at	Stk11	serine/threonine kinase 11	4.54E-03	-1.52
1434627_at	Nrf1	Nuclear respiratory factor 1	1.02E-02	-1.52

Probeset ID	Sirt1-Related	Gene Title	P-value	Fold-Change
1452105_a_at	Tsc2	tuberous sclerosis 2	1.56E-03	-1.56
1448890_at	Klf2	Kruppel-like factor 2 (lung)	1.90E-02	-1.65
1416430_at	Cat	catalase	5.16E-03	-1.65
1450655_at	Pten	phosphatase and tensin homolog	6.86E-03	-1.72
1451791_at	Tfpi	tissue factor pathway inhibitor	9.52E-04	-2.14
1419184_a_at	Fhl2	four and a half LIM domains 2	1.35E-04	-2.15
1435768_at	Arid4b	AT rich interactive domain 4B (RBP1-like)	3.42E-03	-2.66
1448261_at	Cdh1	cadherin 1	2.41E-02	-2.69
1451031_at	Sfrp4	secreted frizzled-related protein 4	1.47E-03	-3.03
1424156_at	Rbl1	retinoblastoma-like 1 (p107)	4.86E-03	-3.14
1449152_at	Cdkn2b	cyclin-dependent kinase inhibitor 2B (p15, inhibits CD	1.43E-03	-3.14
1420765_a_at	Foxp3	forkhead box P3	3.34E-03	-3.28
1433692_at	Nat10	N-acetyltransferase 10	7.16E-03	-3.30
1451218_at	Edem1	ER degradation enhancer, mannosidase alpha-like 1	7.74E-05	-3.43
1451977_at	Dyrk1a	dual-specificity tyrosine-(Y)-phosphorylation regulate	6.74E-03	-3.46
1449335_at	Timp3	tissue inhibitor of metalloproteinase 3	2.41E-06	-3.50
1419105_at	Nr1h4	nuclear receptor subfamily 1, group H, member 4	2.72E-02	-3.51
1448239_at	Hmox1	heme oxygenase (decycling) 1	1.48E-02	-4.05
1422833_at	Foxa2	forkhead box A2	7.60E-05	-4.10
1423023_at	Sfrp5	secreted frizzled-related sequence protein 5	9.94E-04	-4.21
1457721_at	Ppara	peroxisome proliferator activated receptor alpha	2.96E-03	-4.29
1418102_at	Hes1	hairy and enhancer of split 1 (Drosophila)	1.36E-03	-4.31
1450140_a_at	Cdkn2a	cyclin-dependent kinase inhibitor 2A	3.25E-05	-4.43
1421679_a_at	Cdkn1a	cyclin-dependent kinase inhibitor 1A (P21)	4.27E-04	-4.74
1448610_a_at	Sod2	superoxide dismutase 2, mitochondrial	1.47E-02	-4.85
1420715_a_at	Pparg	peroxisome proliferator activated receptor gamma	3.71E-03	-4.94
1457578_at	Ppargc1b	peroxisome proliferative activated receptor, gamma, c	1.04E-02	-5.03
1418640_at	Sirt1	sirtuin 1 (silent mating type information regulation 2, h	1.06E-02	-5.08
1450330_at	Il10	interleukin 10	4.91E-03	-5.47
1420360_at	Dkk1	dickkopf homolog 1 (Xenopus laevis)	4.00E-04	-5.58

Supplementary Table 2. The primers for mouse SIRT1 promoter amplification

Primer	Sequence(5' to 3')
PGL3-SIRT1P-F	ACACTCGAGCATGTGCAATTGAGCCCTAA
PGL3-SIRT1P-R	ACCAAGCTTGGTGGTTCAAGTTTGCGATG
SIRT1P-IRF-MR	CCTCGTCTCAAAAACTTTAAAACGATAGACCATGA
SIRT1P-IRF-MF	GTCTATCGTTTTAAAGTTTTTGAGACGAGGTCTTA

Supplementary Table 3. The primers for real-time PCR

Primer	Sequence(5' to 3')
Adam10-F	TCATCAAGACTCGTGGTGGC
Adam10-R	ATGCTTCTCTGGATGTGCCC
Akt2-F	GATGGTCGCCAACAGTCTGA
Akt2-R	CTCCTTGCGCAGGATCTTCA
Arid4b-F	CACACCAGAGTCGCCTTCTT
Arid4b-R	TGCTACTGCTGGAACTCACG
Bdnf-F	AACGTCCACGGACAAGGCAACTT
Bdnf-R	CCAAAGGCACTTGACTGCTGAGCA
Cat-F	AGGCTCAGCTGACACAGTTC
Cat-R	ATGGAGAGACTCGGGACGAA
Cdk1-F	AAGTGTGGCCAGAAGTCGAG
Cdk1-R	TCGTCCAGGTTCTTGACGTG
Cdk6-F	AAGGCGCCTATGGGAAGGTGTT
Cdk6-R	AGCTTGGTTTCTCTGTCCGTCCGT
Ccnd1-F	TGTGCTGCGAAGTGGAGACCAT
Ccnd1-R	ACTTCTGCTCCTCACAGACCTCCA
Ccnd2-F	GCCAAGATCACCCACACTGA
Ccnd2-R	GCGTTATGCTGCTCTTGACG
Dyrk1a-F	TCAGTGGTGCCAATGAGGTC
Dyrk1a-R	CTTGCTTTCGGTGCTTGGTC
Edem1-F	ACACCTGGATTGACTCGCTG
Edem1-R	GGCCTGGAGTTGCCAGTTAT
Ezh2-F	TGTCGGTGCAAAGCACAATG
Ezh2-R	ACTGTCCCAATGGTCAGCAG
Fhl2-F	TGAGAAGCAGTATGCCCTGC
Fhl2-R	ACTCATCCCGTGCTGTGAAG
Fos-F	TGTGGCCTCCCTGGATTTGACT

Fos-R	AGCTCCACGTTGCTGATGCTCT
Fn1-F	CCGGTGGCTGTCAGTCAGA
Fn1-R	CCGTTCCCACTGCTGATTTATC
Hes1-F	CAACACGACACCGGACAAAC
Hes1-R	CGGAGGTGCTTCACAGTCAT
Hmox1-F	TCCAAGCCGAGAATGCTGAG
Hmox1-R	GGCGTGCAAGGGATGATTTC
Hnrnpk-F	TCAGAGTCTGGCAGGAGGAA
Hnrnpk-R	GCACGTCCTTTGATGGGAGA
Igf1-F	ACCTCAGACAGGCATTGTGG
Igf1-R	TCTTGGGCATGTCAGTGTGG
Klf2-F	ACAATTCCCAGTCAGAGGCG
Klf2-R	CAGTGCCACGGGTCTGTAAT
Lama3-F	CGCTCGGGCTCCTATTCATT
Lama3-R	GGTAATTCCAGGCAGGCAGT
Mmp2-F	TTTGCTCGGGCCTTAAAAGTAT
Mmp2-R	CCATCAAACGGGTATCCATCTC
Mmp-9-F	CGGACCCGAAGCGGACAT
Mmp-9-R	GGGGCACCATTTGAGTTT
Nat10-F	ACAACTCCGTCAGCAGAGTG
Nat10-R	CCAGGCACAGCAGGTCATTA
Nr1h2-F	ACGCTACAACCACGAGACAG
Nr1h2-R	TGATGGCGATAAGCAAGGCA
Nrf1-F	ATGTCCGCACAGAAGAGCAA
Nrf1-R	TGGTGGCCTGAGTTTGTGTT
Pparg-F	ATTCTGGCCCACCAACTTCGG
Pparg-R	TGGAAGCCTGATGCTTTATCCCCA
Rbl1-F	TCTCCACACAAGAACGGAGC
Rbl1-R	AGAGACGTTTGGCAGGTGAG

Rock1-F	CGGGATCCCAAATCGGAAGT
Rock1-R	GCACCTCTGCCGATTACCTT
Rock2-F	ACCTGTCAAGCGTGGTAGTG
Rock2-R	GTGACCGCAGCTGTTCAATG
Sfrp4-F	CCAATTCCTCCTGCCAGTGT
Sfrp4-R	GCATCATCCTTGAACGCCAC
Sirt1-F	TACCTTGGAGCAGGTTGCAG
Sirt1-R	GCTTCATGATGGCAAGTGGC
Stat3-F	ACCAACGACCTGCAGCAATA
Stat3-R	ACACTCCGAGGTCAGATCCA
Stk11-F	TCACCATCCCTTGTGACTGC
Stk11-R	AACCAGCTGTGCTGCCTAAT
Tfpi-F	GCTTAGCCTTGTTCCCGAGT
Tfpi-R	CGGTTCTCGTTCCCTTCACA
Timp3-F	GGCCTCAATTACCGCTACCA
Timp3-R	ATGCAGGCGTAGTGTTTGGA
Tsc2-F	CAGTGTCGACCAGCTGTCTT
Tsc2-R	TCACGCTGTCTGGTCTTGTC
